# Assessing Label Stability in Oligopeptide-Modified Polymer Filament for Advanced Materials: Ultraviolet Exposure and Biodegradation Study

**DOI:** 10.1021/acssuschemeng.5c04602

**Published:** 2025-09-02

**Authors:** Joanna Rydz, Khadar Duale, Marta Musioł, Henryk Janeczek, Anna Hercog, Andrzej Marcinkowski, Kristof Molnar, Frederick C. Michel, Michael Klingman, Maria Letizia Focarete, Judit E. Puskas, Przemysław Mielczarek, Piotr Suder, Miroslawa El Fray, Konrad Walkowiak, Joanna Rokicka, Malwina Niedźwiedź, Alexander Grundmann, Simon T. Kaysser, Sönke Detjen, Brian Johnston, Iza Radecka, Vinodh Kannappan, Marek Kowalczuk

**Affiliations:** § 111480 Centre of Polymer and Carbon Materials Polish Academy of Sciences, M. Curie-Skłodowskiej 34, Zabrze 41-819, Poland; # SPIN-Lab Centre for Microscopic Research on Matter, University of Silesia in Katowice, 75 Pułku Piechoty 1A, Chorzów 41-500, Poland; ◆ Department of Food, Agricultural and Biological Engineering, College of Food, Agricultural, and Environmental Sciences, 2647 The Ohio State University, 1680 Madison Avenue, Wooster, Ohio 44691, United States; ∥ Department of Biophysics and Radiation Biology, Faculty of Medicine, 37637 Semmelweis University, Nagyvarad ter 4, Budapest 1089, Hungary; ¥ Department of Food, Agricultural and Biological Engineering, Ohio Agricultural Research and Development Center, The Ohio State University, 1680 Madison Avenue, Wooster, Ohio 44691, United States; ⊥ Department of Chemistry “Giacomo Ciamician” and INSTM UdR of Bologna, University of Bologna, via Gobetti 85, Bologna 40129, Italy; ‡ Department of Analytical Chemistry and Biochemistry, AGH University of Krakow, A. Mickiewicza 30, Kraków 30-059, Poland; † Laboratory of Proteomics and Mass Spectrometry, Maj Institute of Pharmacology, Polish Academy of Sciences, Smętna 12, Kraków 31-343, Poland; ∇ Department of Polymer and Biomaterials Science, West Pomeranian University of Technology in Szczecin, Al. Piastów 45, Szczecin 70-311, Poland; ○ CompriseTec GmbH, Rödingsmarkt 20, Hamburg 20459, Germany; ± School of Life Sciences, Faculty of Science and Engineering, 8695 University of Wolverhampton, Wulfruna St., Wolverhampton WV1 1LY, U.K.; ! Research Institute in Healthcare Science, Faculty of Science and Engineering, University of Wolverhampton, Wolverhampton WV1 1LY, U.K.

**Keywords:** poly(1,4-butylene adipate-*co*-1,4-butylene terephthalate), polylactide, filament, labeling, oligopeptide, UV irradiation, aerobic composting, anaerobic digestion

## Abstract

This study explores the integration of triglycine, an oligopeptide, as a green molecular marker in 3D-printed poly­(1,4-butylene adipate-*co*-1,4-butylene terephthalate)/polylactide (PBAT/PLA)-based specimens with different printing temperatures to enhance the traceability of (bio)­degradable polymers. This approach supports the advancement of sustainable materials by enabling the identification of the material origin and degradation processes. The research assesses the behavior of the labeled polymer under UV exposure, evaluating the stability of the oligopeptide marker to ensure that the information remains retrievable even after exposure to environmental stressors. In addition, their behavior during aerobic composting, as well as anaerobic digestion, is investigated to promote environmentally friendly practices. This study employed an extraction procedure to isolate and retrieve encoded information, which was then analyzed using a mass spectrometry method, ESI/TIMS-Q-TOF. This makes it possible to determine the sequence of the oligopeptide and compare it with the previously used MALDI-TOF/TOF mass spectrometry procedure. Cytotoxicity studies were also conducted to assess the potential hazards associated with PBAT/PLA-based specimens, considering their potential biomedical applications. The PBAT/PLA-based specimens demonstrated good oligopeptide stability, enabling effective retrieval of recorded information from the green polymer/oligopeptide system even after UV exposure. UV irradiation affected cold crystallization temperature and melting temperature and caused self-chain/cross-linking of the PBAT/PLA-based specimens. In general, the analyses show that specimens printed at a higher temperature (190 °C) have a higher degradation rate than those printed at a lower temperature (155 °C). This phenomenon was attributed to the higher porosity and increased water permeability of the specimens printed at 190 °C, compared to those printed at 155 °C, which is likely due to the greater phase separation and reduced miscibility in the former.

## Graphical abstract

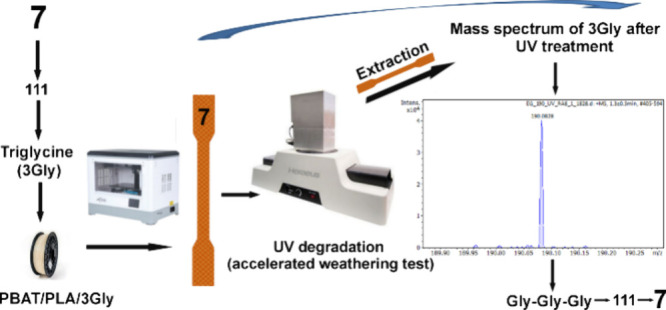

## Introduction

Bioplastics, derived from renewable biomass sources such as maize starch, sugar cane, or potato starch, offer a promising alternative to traditional, fossil-based plastics. They can lower the carbon footprint; in addition, some bioplastics are (bio)­degradable and can be composted, reducing plastic waste and improving sustainability. Their applications range from packaging materials to textiles, medical devices, and construction materials, offering a viable solution for a more eco-friendly future.^1^

A notable example of a modern bioplastic that has gained a lot of interest in recent years is Ecovio—poly­(1,4-butylene adipate-*co*-1,4-butylene terephthalate)/polylactide­(PBAT/PLA). This is a (bio)­degradable aliphatic-aromatic copolyester blend with a wide range of applications in the medical and packaging industries. Its unique properties make it an ideal choice for medical and implantable devices such as surgical meshes, wound dressings, and disposable products, as well as packaging materials. Furthermore, its (bio)­degradable nature ensures that it can be easily composted in both industrial and home composting facilities, reducing waste and environmental impact.^2^ Although Ecovio has outstanding physical and mechanical properties, as PBAT reduces PLA’s high stiffness and improves tear strength, research on Ecovio in three-dimensional (3D) printing is limited. The limited research is due to the difficulties arising from optimizing processing conditions and the specific layering techniques required to produce it.^3,4^

Molecular labeling of (bio)­degradable polymers enables the incorporation of labels into polymers, allowing for the personalization of the material, anticounterfeiting measures, and authentication confirmation. This labeling process also enables the recording and storage of information, which can be crucial in forensic engineering applications, facilitating the identification of materials in various contexts, including criminal investigations.^5^ Storage of information in polymers is an emerging field that has inspired the development of new synthetic materials with encoded information, often mimicking biological codes such as DNA. Researchers have synthesized monodisperse, digitally encoded polymers with higher information storage density than DNA. In addition, various methods for decoding and reading this information have been explored, including nanopore technology and chiral liquid crystal polymers. These developments open up new possibilities for labeling and tracking products, such as identifying the expiry date or tracing the life cycle of a product.^6–8^ Such materials also fit with the new strategy “Advanced Materials for Industrial Leadership” launched by the European Commission to promote the development and use of advanced materials in various industrial sectors.^9,10^ Achieving this, however, requires precise control over comonomer sequences and the development of analytical methods enabling the reading of the encoded information, which is particularly challenging for high-molar-mass copolymers.^11^ Mass spectrometry (MS) is a widely used method for decoding the sequence of oligomer mixtures, allowing for precise characterization of the composition of a single submillimeter spot on the immobilized matrix without separation. This technique has been successfully applied to the sequencing of oligohydroxyalkanoate copolymers, providing information about their molecular structure, and has also been adopted for routine analysis of poly­(3-hydroxybutyrate*-co*-3-hydroxyvalerate) (PHBV).^12^ In this context, in our recent work, an oligopeptide was introduced into a polymer film through pressing, which allows its subsequent extraction from the polymer matrix and analysis using a matrix-assisted laser desorption ionization (MALDI-TOF/TOF; TOF—time-of-flight) MS. The resulting data were then interpreted using BioTools software to decrypt the binary-encoded information stored in the oligopeptide sequence, allowing it to be read.^5^ The integration of bioactive molecules into (bio)­degradable polymer materials enables the development of smart biomaterials with specific properties. The development of polymers with stored information at the molecular level has vast potential applications, including long-term data archiving, anticounterfeiting systems, molecular cryptography, and identifying recyclable or compostable plastics.

In a recent study, after establishing appropriate mixing and processing conditions, neat Ecovio and Ecovio with the addition of 0.2 wt % monodisperse oligopeptide (triglycine) as a marker, a labeled filament for additive manufacturing was obtained, from which dumbbell-shaped specimens were then printed.^13^ This work discusses in detail the issues related to the matrix compatibility and stability of triglycine during processing. Specific applications of polymers depend on their durability, i.e., resistance to degradation phenomena such as photo-oxidation, hydrolytic degradation, or biodegradation.^14^ One of the challenges associated with medical devices and their packaging made of (bio)­degradable polymers is the need for sterilization. The packaging must not be damaged during sterilization and must provide physical protection against potential external damage during transport and storage. Likewise, the labeling should not be damaged during the sterilization and aging processes, allowing it to be retrieved even at the end-of-life stage.

In the case of PLA, the main photodecomposition pathway is the photolysis reaction that leads to the breaking of C–O bonds in the macromolecule backbone and the photooxidative mechanism that leads to the formation of hydroperoxide derivative and its subsequent degradation to compounds containing carboxylic acid and diketone end groups. In addition, photolysis of the diketone can lead to homolytic cleavage of the C–C bond between the two carbonyl groups, resulting in the formation of two carbonyl radicals. The Norrish type II photo cleavage can also occur at ester and ethylidene groups adjacent to the ester oxygen. It was observed that the photodegradability of PLA chains in the crystalline region is lower than in the amorphous regions.^14–17^ Norrish type I and II chain scission pathways are the main photodegradation pathways of PBAT. There may also be a chain cross-linking caused by free radicals generated during the Norrish type I reaction.^14,18^

It is important to select an appropriate biobased material for molecular labeling of commercially available (bio)­degradable copolymers. In this study, a triglycine was selected as a green molecular marker with a monodisperse sequence, and a PBAT/PLA-based filament was prepared with its addition, from which dumbbell-shaped specimens were then printed. The selected marker containing binary information chemically encoded in the backbone was characterized for use in the molecular labeling of packaging for medical applications and other special products suitable for packaging materials. The study proposes a straightforward method to overcome the difficulties of molecular labeling for (bio)­degradable polymers. The primary objective is to label each bioplastic with a unique oligopeptide sequence that corresponds to the encoded information, enabling specific identification and tracking of the polymers. The incorporation of oligopeptides into polymer materials enables the introduction of binary-encoded information that can be extracted and decoded using mass spectrometry. This approach enables the labeling of (bio)­degradable polymer materials, making it possible to track their origin, composition, and degradation pathways, which is crucial for the development of sustainable and environmentally friendly materials. Furthermore, the properties of the labeled polymer during composting and anaerobic digestion were also investigated. In particular, its behavior during ultraviolet (UV) exposure, to assess the stability of the label (triglycine) contained in the polymer matrix, so that the recorded information from the (bio)­degradable polymer/triglycine system could also be retrieved after UV exposure (Scheme 1).

**Scheme 1:**
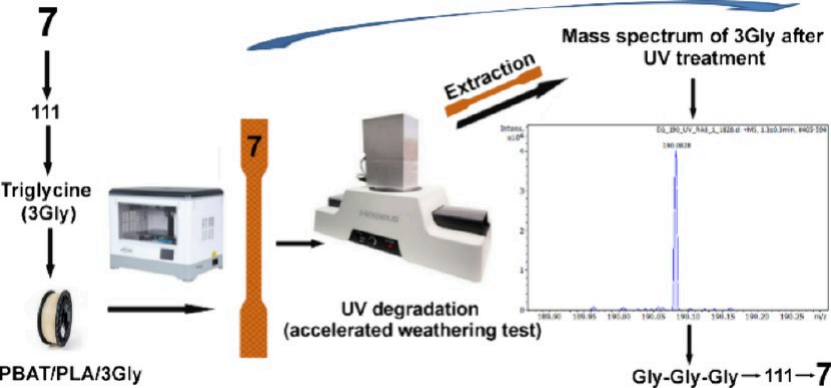
Schematic Presentation of the Idea of Labeling^a^

a Model oligopeptide—triglycine (Gly-Gly-Gly) as binary notation 111 and Arabic notation 7 was used for labeling.

The determination of the molar mass and dispersity of polymers (by gel permeation chromatography, GPC) is intended to ensure future processability and selection of the appropriate sterilization method. The PBAT/PLA-based specimens were also subsequently characterized using a proton nuclear magnetic resonance (^1^H NMR), differential scanning calorimetry (DSC), and thermogravimetric analysis (TGA). Moreover, the morphology of the 3D-printed specimens was investigated by using optical microscopy and scanning electron microscopy (SEM). The (bio)­degradable polymers with their additive (in this case, triglycine) were also assessed for their cytotoxicity. The viability of cell lines of MDA-MB231 breast cancer, HEK293 human embryonic kidney, MSTO human mesothelioma cancer, and A549 human lung cancer was assessed by microscopic observation, and the metabolic activity of the populations was measured by an MTT assay after 48 h exposure to investigate cell proliferation.

The study presents several aspects of scientific novelty and merit, such as (i) molecular data storage: the use of triglycine as a molecular label for PBAT/PLA in the context of data storage is a novel approach. Encoding and decoding numerical information via mass spectrometry (ESI/TIMS-Q-TOF) demonstrates high sensitivity in molecular-level information storage. (ii) Advanced mass spectrometry analysis: the comparison of ESI/TIMS-Q-TOF to MALDI-TOF/TOF highlights the strengths and limitations of both methods in identifying short oligopeptides like triglycine, particularly in terms of ion mobility, which improves the identification accuracy. (iii) UV-induced structural changes: the study comprehensively investigates how UV exposure influences the thermal stability and degradation patterns of PBAT/PLA. It details self-chain/cross-linking mechanisms, improving the understanding of the polymer’s stability under environmental conditions. (iv) Cytotoxicity and biocompatibility: the findings on cytotoxicity at processing temperatures above 120 °C highlight a crucial limitation for potential applications in biomedical and food-contact materials.

## Experimental Section

### Materials

Triglycine (GGG, 3Gly, C_6_H_15_N_3_O_6_) from Sigma-Aldrich, Taufkirchen, Germany, with a purity of ≥ 98% and the relative molar mass *M*_*r*_ = 189.17 g mol^–1^ (binary notation: 111, Arabic notation: 7) was used as a model oligopeptide for labeling as received. Poly­(1,4-butylene adipate-*co*-1,4-butylene terephthalate)/polylactide (PBAT/PLA, commercial blend Ecovio F Mulch C2311) pellet containing 47 mol % of aromatic segments with 25 mol % of PLA (determined by ^1^H NMR)^19^ from BASF Ludwigshafen, Germany, was used for the filament preparation. Microcrystalline cellulose with an average particle size of 50 μm provided by ThermoScientific Chemicals (Waltham, MA, USA) was used as a positive control in the anaerobic digestion experiment.

### PBAT/PLA-Based Specimens’ Preparation

3D-printed PBAT/PLA-based specimens with two printing temperatures, 155 and 190 °C, as well as with and without triglycine (see Table 1) were fabricated using a material extrusion (MEX) 3D printer (Artillery Sidewinder X1, Shenzhen Yuntu Chuangzhi Technology Co.; Ltd.; Shenzhen, China) from filaments produced with a single-screw extruder (Extrudex, Mühlacker, Germany) according to ISO 527:2019 standard^20^ (the exact procedure of preparing the composites is described in ref 13). The temperature profile in the direction was as follows: 100–105–110–115–120 °C. Dumbbell-shaped specimens of the 1BA type with geometry according to ISO 178:2019 standard^21^ with dimensions *x* = 12.5 mm (width), *y* = 80 mm (length), and *z* = 2 mm (thickness, Figure 1) were printed according to the procedure presented in ref 13.

**Table 1:** PBAT/PLA-Based Specimens Were Fabricated by MEX

specimen name	nozzle temperature [°C]	triglycine [wt %]
PBAT/PLA_155	155	
PBAT/PLA/3Gly_155	155	0.2
PBAT/PLA_190	190	
PBAT/PLA/3Gly_190	190	0.2

**Figure 1:**
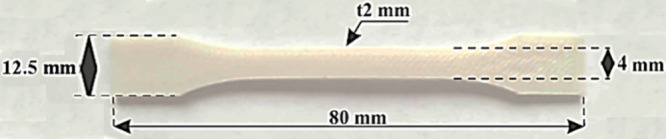
Shape and dimensions of PBAT/PLA-based specimens.

### Ultraviolet Exposure Experiments

The PBAT/PLA-based specimens were UV exposed under a 120 W/cm quartz Fusion UV lamp compatible electrodeless 6″ H type with spectral distribution 200–450 nm parallel to the specimen with a Heraeus Noblelight Fusion UV unit equipped with an LC6B benchtop conveyor with F300S system (Heraeus Noblelight America LLC, Gaithersburg, MD, US). The dumbbell-shaped specimens were placed at a distance of 5 cm from the irradiator face and exposed to UV light 3 times per side in air with belt speeds of 1 m/min under a total UV dose of 1920 kJ/m^2^.

### Aerobic Composting Experiments

Standardized laboratory-scale composting was conducted using a reactor system to investigate the biodegradability of the PBAT/PLA-based specimens during composting (tested materials; see Table 2). The degree of biodegradation was calculated by measuring the average amount of carbon (C, in particular carbon dioxide, CO_2_) mineralized from each reactor and subtracting the average amount of carbon mineralized from the blank reactor, and then dividing this by the total amount of carbon in the specimen added to each reactor.^22^ Three reactors containing only compost were used as the blanks.

**Table 2:** Tested Materials for Aerobic Composting Experiment

specimens	mass of specimens [g]	C content in the specimens from elemental analysis [wt %]	C content in degraded specimens [g]
PBAT/PLA_155	8.1	50.2 ± 0.8	4.07
PBAT/PLA/3Gly_155	5.99	56.3 ± 1.6	3.37
PBAT/PLA_190	8.5	47.6 ± 1.5	4.05
PBAT/PLA/3Gly_190	6.89	51.9 ± 1.3	4.05
blanks (negative control, compost without specimens)	0		
toilet paper (positive control)	10.1	39.9 ± 0.6	3.83

The PBAT/PLA-based specimens were tested under simulated composting conditions from mid-July to mid-October 2023 for 97 days, following the protocol outlined in ASTM D5338–98(2003),^23^ equivalent to ISO 14852:2021.^24^ This test measured the degree and rate of conversion of carbon to CO_2_ under conditions that mimic the moisture, temperature, and aeration conditions at an industrial composting facility.^22^ The mass of the tested materials varied from 6.0 to 8.5 g (4 to 6 specimens of each tested material). Each material was mixed with 502 ± 2 g of mature compost with a moisture content of 79 ± 0.2%, so each vessel contained approximately 509 ± 1 g of the substance on a wet-mass basis.

The compost inoculum was from a full-scale compost curing pile made from a turned windrow on a concrete surface at the Ohio Agricultural Research and Development Center, the Ohio State University in Wooster, OH, US. The compost consisted of a mixture of dairy manure and sawdust from deciduous trees as described in ref 25. The compost and test materials were placed together in a reactor—vessels with a working capacity of 4 L, each 30 cm long and 15 cm in diameter, constructed from poly­(vinyl chloride) (PVC) pipe and placed in an incubator (BioCold Environmental Inc.; MO, US) at 56 ± 0.5 °C. The airflow rate to each reactor was controlled using a flow restrictor at a flow rate of 100 mL/min to ensure aerobic conditions. To prevent drying during the experiment, the air was saturated by bubbling it through bottles containing deionized water at the incubator temperature. Reactors were aerated from below, and the air leaving the reactor vessels was directed through 250 mL flasks in a separate water bath maintained at 8 °C to reduce the moisture content of the off-gas and prevent downstream condensation. The percentage of CO_2_ in the off-gases was measured using an infrared gas analyzer (Vaisala model GMT 220, range 0–20%). The CO_2_ data were automatically recorded every 1 h for each reactor with a Campbell Scientific model 23XL (Logan, UT, US) data logger. Additionally, each reactor was equipped with a K-type thermocouple to monitor the temperatures of the compost mixture near the reactor’s midpoint, and automatically recorded along with ambient temperatures and pressures every 12 min.^26,27^

### Anaerobic Digestion Experiments

Anaerobic digestion experiments at a constant temperature of 37 ± 1 °C in a high-solids batch anaerobic digestion were performed in triplicate for 3 months (99 days) from early March to early June 2024, according to the standard protocol described in ASTM D5511–02 ^22^ equivalent to ISO 15985:2014.^28^ During incubation under controlled anaerobic conditions, the conversion of PBAT/PLA-based specimens to CO_2_ and methane (CH_4_) was measured. Specimens were exposed to an active wet methanogenic inoculum obtained in March 2024 from a full-scale (3000 m^3^) anaerobic digestion system located on the Wooster campus of Ohio State University that treats municipal and commercial food processing waste.^22,29^ A blank test reactor contained only 200 g of active methanogenic inoculum, and a positive control contained 200 g of inoculum plus cellulose. Treatment reactors contained four PBAT/PLA-based specimens (with a total mass of 5.7 ± 0.7 g) of each type of material (PBAT/PLA_155, PBAT/PLA/3Gly_155, PBAT/PLA_190, PBAT/PLA/3Gly_190, see Tables 1 and 2) along with 200 g of active methanogenic inoculum and mixed thoroughly. These mixtures were then placed in 250 mL (working volume) laboratory-scale batch reactors that were incubated in a temperature-controlled room at 37 ± 1 °C and shaken at 115 rpm on an orbital shaker. To capture the released gases, each reactor was equipped with a gas collection bag. To maintain an anaerobic atmosphere, each reactor was initially purged with nitrogen to displace the residual air. Bags were removed when full, and the volumes were measured by water displacement at room temperature.

The degree of biodegradation was calculated using the total volume of CO_2_ and CH_4_ produced over the 99 days of the experiment from each treatment, and converting the volumes to moles of carbon using the ideal gas law (eq 1).

n=PV/RT 1

where *n* is the amount of substance [mol], *P* is the pressure [Pa], *V* is the volume [m^3^], *R* is the gas constant, 8.314 m^3^·Pa·K^–1^·mol^–1^, and *T* is the temperature [K] at which the volume was measured (310.15 K = 37 °C). The amount of carbon obtained was multiplied by atomic mass (*A*_*r*_) = 12 g·mol^–1^ to determine the mass (g) of carbon emitted during each treatment. The amount of carbon produced by the negative control was then subtracted from the amount of carbon for each treatment. This correction took into account background emissions unrelated to the tested PBAT/PLA-based specimens. Finally, the amount of carbon emitted from the specimens was divided by the total mass of carbon in the tested specimens. These calculations considered the percentages of carbon in the PBAT/PLA-based specimens that were converted to CO_2_ and CH_4_ during the experiment. After completing the experiment, all specimens were separated from the sludge and meticulously cleaned, dried, and weighed. The washing process was performed using distilled water and ethanol.

### Methods

#### Nuclear Magnetic Resonance (NMR) Spectroscopy

^1^H NMR spectra of PBAT/PLA-based specimens were recorded using a Bruker Advance spectrometer operating at 600 MHz (Bruker BioSpin GmbH, Rheinstetten, Germany) with Bruker TOPSPIN 2.0 software using CDCl_3_ as the solvent and tetramethylsilane (TMS) as the internal standard. Spectra were obtained with 64 scans, an 11 μs pulse width, and a 2.66 s acquisition time.

#### Gel Permeation Chromatography (GPC) Analysis

The molar mass and molar-mass dispersity of the PBAT/PLA-based specimens were analyzed using a GPC with mixed-bed columns (a set of two PL-gel 5 μm MIXED-C ultrahigh efficiency columns, Polymer Laboratories, and a linear range of mass-average molar mass (*M*_*w*_) = 200–2000000 g·mol^–1^) and chloroform as the eluent at 35 °C with a flow rate of 1 mL·min^–1^. Ten μL of 0.3% m·V^–1^ specimen solution was injected into the system. A Nexera HPLC/UHPLC pump, LC-40D XR (Shimadzu, Kyoto, Japan), and a Shodex Refractive index detector, RI-101 (Shoko Scientific Co., Ltd., Yokohama, Japan), were used to determine the molar masses, which were calculated using a universal calibration curve generated from polystyrene standards (EasiCal preprepared calibration kits, Polymer Laboratories, Shropshire, UK) with narrow molar-mass dispersity and OmniSEC 5.0 (Viscotek, Malvern Panalytical, Malvern, UK) software.

#### Differential Scanning Calorimetry (DSC)

Thermal characteristics of the PBAT/PLA-based specimens were obtained using a TA-DSC Q2000 apparatus (TA Instruments, Newcastle, DE, US). The instrument was calibrated with a high-purity indium. DSC experiments were performed between −60 and 190 °C with heating and cooling rates of 20 °C·min^–1^. All experiments were performed in a nitrogen atmosphere at a nitrogen flow rate of 50 mL·min^–1^, using standard aluminum sample pans. The glass transition temperature (*T*_*g*_) was taken as the midpoint of the change in heat capacity of the specimen and was measured in the second calorimetric trace (second heating run) for the amorphous samples obtained by rapid cooling (RC) from the melt.

#### Thermogravimetric Analysis (TGA)

Thermal stability of the PBAT/PLA-based specimens was evaluated by the TGA/DSC1 Mettler-Toledo thermal analyzer (Columbus, OH, US) in nitrogen (60 mL·min^–1^) with a heating rate of 10 °C·min^–1^. Mettler-Toledo AG StarSystem SW 9.30, Schwerzenbach, Switzerland, was used to generate a particular set of results.

#### Optical Microscopy Structure Analysis

The morphology of PBAT/PLA-based specimens was observed with a Zeiss optical microscope (Opton-Axioplan, Oberkochen, Germany) equipped with a Nikon Coolpix 4500 color digital camera as well as a modular i4 Infinity microscope with four objectives (LW Scientific, Lawrenceville, GA, US) with a camera attachment and image capture software. Microscopic observations were performed at magnifications of 4, 10, 40, 100, and 120×.

#### Scanning Electron Microscope (SEM)

SEM studies were performed using a Quanta 250 FEG (FEI Company, Hillsboro, OR, US) high-resolution environmental SEM instrument operated at 5 kV acceleration voltages. The PBAT/PLA-based specimens were observed without coating under a low vacuum (80 Pa) by using a secondary electron detector (large field detector).

#### Electrospray Ionization/Trapped Ion Mobility-Quadrupole-Time-of-Flight (ESI/TIMS-Q-TOF) Mass Spectrometry

A 2 mm^3^ sample size of the PBAT/PLA-based specimens with oligopeptide was placed in a tube, and 100 μL of 50% acetonitrile with 0.1% formic acid was added. Then the samples were incubated in an ultrasonic bath for 5 min and centrifuged at 10000 g for 5 min. The supernatant was used for mass spectrometry analysis. Briefly, 0.5 μL of the sample was directly injected into the mass spectrometer by the liquid chromatographer UltiMate3000 (Thermo Scientific, Waltham, MA, US) with a flow rate equal to 3 μL·min^–1^. Both systems (LC-MS; LC, liquid chromatography) were controlled by Hystar software (Bruker Daltonics, Bremen, Germany). The chromatographic system was directly coupled to a timsTOF Pro 2 (Bruker Daltonics, Bremen, Germany) mass spectrometer using an electrospray ion source. The instrument was operated in a positive-ion mode with parameters set as follows: *m*/*z* range 20–1300, ion mobility range (1/K_0_) 0.45–1.45, ramp time 100 ms, drying gas 3 L·min^–1^, drying temp. 150 °C and capillary voltage 1500 V. Mass spectra were externally calibrated with the ESI-L Tuning Mix (Agilent Technologies, Santa Clara, CA, US). A certain discrepancy occurring between the theoretical and experimental masses is within the error limit, as an error below 5 ppm is acceptable (in this case, the largest error was 0.0006 units, i.e., 3 ppm).

#### Elemental Analysis

Elemental analysis was carried out by using the Vario EL III apparatus, Elementar (Langenselbold, Germany).

All experiments and measurements were performed in triplicate.

#### Cytotoxicity Studies

MTT (3-(4,5-dimethylthiazol-2-yl)-2,5-diphenyltetrazolium bromide) cytotoxicity assay was used to assess the cytocompatibility of the PBAT/PLA-based specimens with and without triglycine printed at 155 and 190 °C. Four human cell lines of different origins were used: MDA-MB231 (breast epithelial cancer cell line), A549 (lung epithelial cancer cell line), HEK293 (human embryonic kidney cell line), and MSTO-211H (mesothelioma of lung—fibroblast-like cells). All cell lines were purchased from ATCC (Teddington, UK). Gibco RPMI-1640 cell culture medium, Gibco DMEM, high glucose, GlutaMAX supplement cell culture medium, Gibco Amphotericin B, Gibco penicillin-streptomycin (10000 U/mL), Gibco fetal bovine, MTT, and dimethyl sulfoxide (DMSO) were purchased from Fisher Scientific (Loughborough, UK).

The PBAT/PLA dumbbell-shaped specimens were cut into 1 cm pieces that fit a 24-well plate, and all the above cells were seeded at a density of 1 × 10^4^ cells/well (*n* = 3) with the specimen strips. A control well of cells without any specimen strips was seeded on the same plate. All specimens with the cells were incubated at 37 °C with 5% CO for 72 h. Following incubation, the cells were imaged using EVOS auto2 live cell imager with phase contrast (Thermo Fisher Scientific, UK), and MTT reagent was added to each well and incubated for a further 4 h at 37 °C. The live cells growing on the plate as well as the polymer material formed formazan crystals, which were then solubilized with 300 μL DMSO, and the plate was left on a shaker for 10 min for complete extraction. The resulting-colored solution was transferred (200 μL per sample) to a fresh 96-well microtiter plate, and the absorbance was read at 570 nm using Varioskan Lux (Thermo Fisher Scientific, UK) microplate reader. The percentage of cell viability was calculated by normalizing the absorbance value with a control well. A two-way ANOVA analysis with multiple comparisons was performed to calculate the statistical significance between the different samples in the group.

## Results and Discussion

### Material Characterization and Stability of the Triglycine-Based Molecular Labeling System during UV Exposure

Our latest study^5^ in the molecular labeling field explores a novel approach to storing binary information using an oligopeptide as a molecular marker in (bio)­degradable polymer matrix—a strategy with compelling potential for tracking applications for medical devices and their packaging. This method could enable the tracking of plastic waste by storing critical data within the polymers, allowing for efficient retrieval and reading of the information. Such advancements not only enhance the functionality of (bio)­degradable materials but also contribute to improved waste management and traceability. In this study, the number 7 was encoded as the information in the form of the specific amino acid sequence in a triglycine, which was incorporated into a polymer filament made of a blend of PBAT and PLA (Ecovio).^13^ Its recovery from 3D-printed specimens was successfully carried out through a solvent extraction process and analysis *via* mass spectrometry. This approach enables the retrieval of encoded information irrespective of the polymer’s molar mass or composition, overcoming the challenges typically faced with high-molar-mass copolymers, thereby illustrating a promising route for integrating data storage capabilities into (bio)­degradable materials. The effect of UV exposure of PBAT/PLA-based specimens obtained by 3D printing from a filament extruded at different temperatures was also investigated, as well as the stability of the marker (triglycine) to higher processing temperatures and UV radiation.

Figure 2 shows the theoretical mass spectrum of a standard oligopeptide with the amino acid sequence GGG, which is observed as the [M + H]^+^ peak at *m*/*z* = 190.082 ± 3 ppm. The second peak is the isotopic peak. When analyzing low-molar-mass peptides with a high-resolution spectrometer with low error (the largest error was 0.0006 units), the *m*/*z* value of the parent ion is sufficient to determine the type of oligopeptide based on its molar mass.

**Figure 2:**
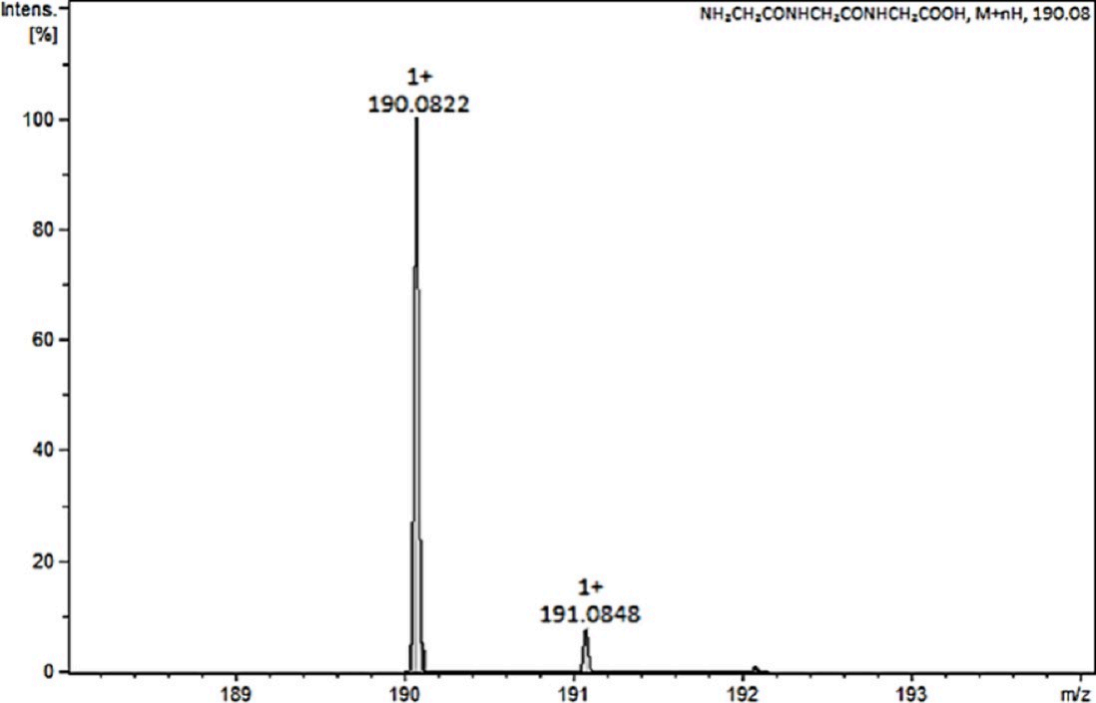
Theoretical mass spectrum of the standard oligopeptide with the amino acid sequence GGG generated by DataAnalysis software (Bruker, Bremen, Germany). The peak at *m*/*z* = 190.082 ± 3 ppm corresponds to a monoisotopic pseudomolecular ion.

In the case of a filament with the oligopeptide in the supernatant after extraction of the polymer matrix, the ion at *m*/*z* = 190.083 ± 3 ppm corresponding to the pseudomolecular ion of the GGG oligopeptide [M + H]^+^ was observed (Figure 3B). Whereas after extraction of the polymer matrix without the oligopeptide marker, no characteristic signal can be observed in the spectrum of the supernatant (Figure 3A) at the *m*/*z* range characteristic of oligopeptide ions.

**Figure 3:**
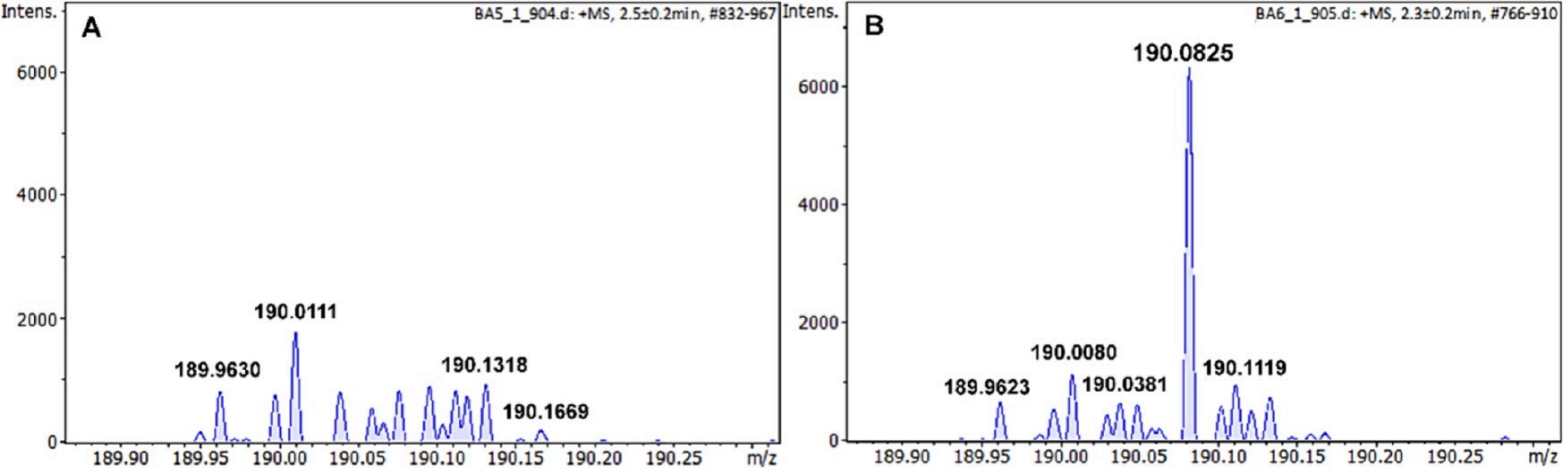
Mass spectra in 50% acetonitrile of the PBAT/PLA-based filament supernatant after extraction of the polymer matrix without (A) and with (B) oligopeptide.

The results of mass spectrometry analysis of the supernatant after extraction of the PBAT/PLA specimen matrix with the oligopeptide obtained at two printing temperatures, 155 and 190 °C, are shown in Figure 4.

**Figure 4:**
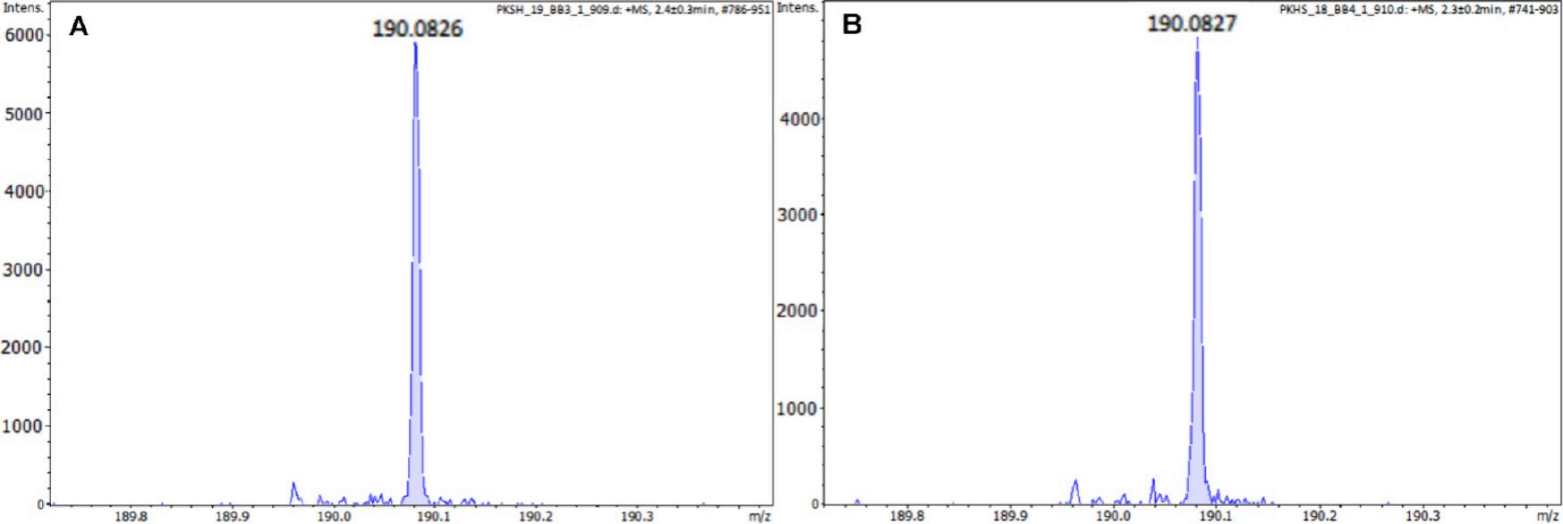
Mass spectra in 50% acetonitrile of oligopeptide with GGG sequence extract from the PBAT/PLA-based specimens were obtained at two printing temperatures, 155 (A) and 190 °C (B).

The ion intensity at *m*/*z* = 190.083 ± 3 ppm decreases slightly as the printing temperature increases, but it is distinctly visible at both printing temperatures.

The mass spectrum of the oligopeptide extracted from the PBAT/PLA-based specimens with the oligopeptide after UV exposure is shown in Figure 5.

**Figure 5:**
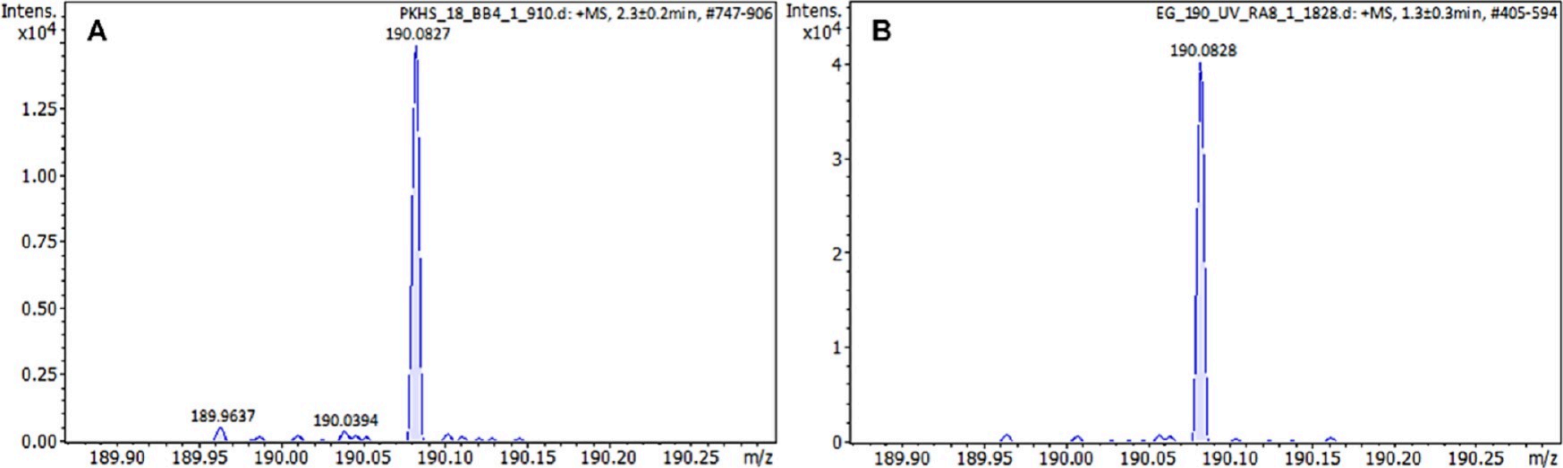
Mass spectra in 50% acetonitrile of oligopeptide with GGG sequence extract from the PBAT/PLA-based specimens obtained at 190 °C before (A) and after the UV exposure experiment (B).

The peak at *m*/*z* = 190.083 ± 3 ppm after the UV exposure experiment has a higher intensity compared to the specimen that was not subjected to UV radiation (see Figure 5). This is probably due to the degradation of the polymer matrix after irradiation, resulting in easier extraction from the degraded material and indicating the good stability of the oligopeptide. Triglycine, as an unbranched oligopeptide, is generally resistant to high temperatures and oxygen conditions (decomposition temperature at 5% mass loss, *T*_5%_ = 251.0 °C and temperature at the maximum decomposition rate, *T*_max_ = 251.2 °C) and the peptide bonds themselves are usually quite strong.^5^ Its resistance may be proven by the fact that it has been found in space in meteorites and comets.^30,31^

The study highlights the high sensitivity of the oligopeptide information in the molecular structure, effectively analyzed by ESI/TIMS-Q-TOF mass spectrometry, but notes a disadvantage compared to the MALDI-TOF/TOF method: ESI/TIMS-Q-TOF lacks software for labeling the amino acid sequence of the fragmented oligopeptide for short amino acid sequences of oligopeptides like GGG, slightly complicating the interpretation of the mass spectrum.

Visual assessment of PBAT/PLA-based specimens obtained by 3D printing from a filament extruded at different temperatures, before and after UV exposure, showed that the specimens partially turned from beige to light brown under the applied irradiation (a total UV dose of 1920 kJ·m^–2^) and were slightly burned and overmelted (Figure 6), which indicates that too much irradiation may destroy the surface of the (bio)­degradable polymer material.

**Figure 6:**
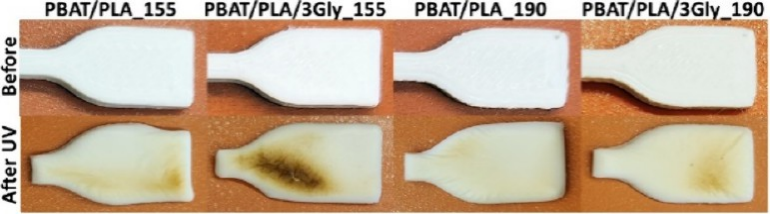
Representative images of PBAT/PLA-based specimens before and after UV exposure.

The images before exposure show a distinct pattern formed during specimens’ 3D printing, while afterward, the surface is uniform and shiny (see also optical microscope images in Figure 10). The images of PBAT/PLA-based specimens after UV exposure show that the UV degradation does not occur evenly across the entire surface of the specimens due to (i) differences in surface topography (surface roughness, with peaks and valleys, that can lead to more rapid degradation at the higher points^32^), (ii) possible differences in the composition of the polymer matrix (phase separation in the blende) and/or (iii) the presence of additives (Ecovio contains talc, which can be unevenly distributed in the polymer matrix^13^).

The mass measurement (data not shown), carried out immediately after the UV exposure experiment and compared with the mass after 5 days of storage of these specimens after UV exposure, showed a slight decrease in mass (≥ 0.2 wt %) due to evaporation of moisture (absorbed from the environment) from the specimens during UV exposure.

The GPC analysis showed a slight (6.5–14%) increase in the *M*_*w*_ of all specimens after UV exposure, which may indicate self-chain-linking (Figure 7).

**Figure 7:**
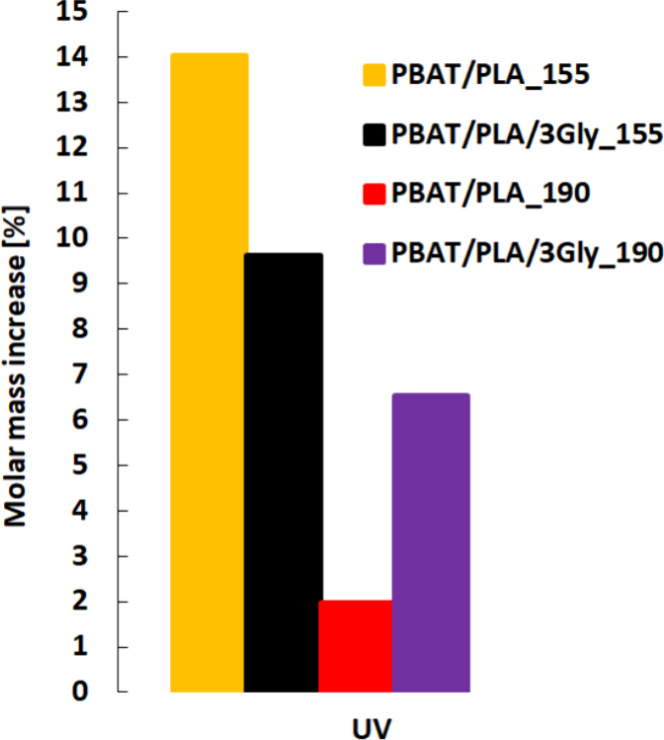
Molar mass increase of PBAT/PLA-based specimens after UV exposure. The molar mass increase is given as a percentage of original *M*_*w*_.

On the basis of the chemical structure of PBAT and its physicochemical properties, it has been considered that self-chain/cross-linking of the virgin polymer is possible, whereas in the case of PLA, it usually degrades, which does not exclude self-chain/cross-linking.^33,34^ Cross-linking, the formation of radiation-induced radicals, plays a crucial role in enhancing the properties of polymers/plastics. This process creates a 3D chain-branching network, resulting in tailoring material properties such as degradability, solubility, gas permeability, and processability.^35^ The irradiation of the polymer can lead to either chain scission or self-cross-linking reactions, which occur primarily in the amorphous region, and some may occur in the interphase between the amorphous and crystalline regions. The effectiveness of chain self-cross-linking depends on various factors, including the type of polymer, irradiation parameters, and environmental conditions, such as the presence or absence of oxygen and temperature.^33,36^

Specimens printed at a temperature of 155 °C were self-chained/cross-linked to a greater extent (the highest molar mass increase) than those printed at 190 °C (see Figure 7). As the self-chain/cross-linking is to a small extent, it does not significantly change the solubility of PBAT/PLA-based specimens. It was previously observed that, in contrast to the PBAT/PLA-based specimens exposed to a UV dose of 1920 kJ·m^–2^ in this experiment, which caused an increase in molar mass with a simultaneous significant increase in dispersion, pure PLA exposed to a UV dose of 2214 kJ·m^–2^, however, at a much longer exposure time (8 h compared to 4.5 s in this experiment), decreased the molar mass by about 90% of the original molar mass of PLA.^34^

In order to confirm self-chaining/cross-linking after UV exposure, the thermal behavior of the PBAT/PLA-based specimens was investigated (Table 3).

**Table 3:** Calorimetric Parameters of PBAT/PLA-Based Specimens before and after UV Exposure, as well as after 77 and 97 Days of Aerobic Composting and after 30, 58, and 99 Days of Anaerobic Digestion^a^

	first heating run				second heating run after RC				cooling run (third run)			
specimen	*T*_*cc*_ [°C]*	ΔΗ_*cc*_ [J·g^–1^]	*T*_*m1*_ [°C]*	*T*_*m2*_ [°C]	Δ*H*_*m*_ [J·g^–1^]	*T*_*g*1_/*T*_*g*2_ [°C]	*T*_*m1*_ [°C]*	*T*_*m2*_ [°C]	Δ*H*_*m*_ [J·g^–1^]	*T*_*g*1_/*T*_*g*2_ [°C]	*T*_*c*_ [°C]*	ΔΗ_*c*_ [J·g^–1^]
Before Degradation												
PBAT/PLA_155	61.5/92.5	0.51/4.69	131.7	154.4	4.91	–30.0/59.1	130.8	155.6	9.16/0.63	–37.4/53.4	98.2	10.05
PBAT/PLA/3Gly_155	60.9/93.9	0.52/5.98	133.8	153.6	5.7/1.18	–32.3/59.6	130.8	155.6	6.54/0.73	–38.9/52.5	97.6	8.48
PBAT/PLA_190	61.9/89.2	0.39/2.52	132.8	151.7	6.53	–30.7/57.6.3	130.3	154.7	6.05/0.86	–36.5/55.2	98.4	8.69
PBAT/PLA/3Gly_190	64.9/97.8	0.37/5.65	132.2	154.3	6.08/0.95	–32.7/59.2	130.6	156.0	6.74/0.76	–38.1/52.8	98.4	9.53
After UV Exposure												
PBAT/PLA_155	-	-	134.1	155.0	8.39	–31.3/59.5	130.7	152.1	8.07	–39.4/54.5	96.3	8.16
PBAT/PLA/3Gly_155	-	-	93.0/140.3	154.3	1.91/10.19	–29.3/61.3	131.3	153.3	8.16	–38.7/48.2	96.1	8.86
PBAT/PLA_190	-	-	125.6	151.9	5.33	–29.6/57.5	125.6	151.5	6.43/0.95	–38.0/53.1	99.2	7.18
PBAT/PLA/3Gly_190	-	-	130.3	152.7	5.78	–31.3/59.9	130.1	151.8	5.03	–37.9/48.5	99.2	5.98
After 77 Days of Aerobic Composting												
PBAT/PLA_155	-	-	134.3		13.06	–27.0/-	135.5	-	2.10	–42	107.6	10.43
PBAT/PLA/3Gly_155	-	-	135.0	145.1	16.53	–27.9/-	136.4	-	11.96	–32.5	106.8	10.46
PBAT/PLA_190	-	-	134.0	151.4	12.85	–29.3/-	132.5	-	11.34	–34.9	109.5	11.36
PBAT/PLA/3Gly_190	-	-	136.2	-	19.41	–31.3/-	135.9	-	11.41	–36.1	107.3	13.33
After 97 Days of Aerobic Composting												
PBAT/PLA_155	-	-	122.9	144.0	17.84	–27.6/-	122.5	140.7	11.74	–33.5	115.7	13.14
PBAT/PLA/3Gly_155	-	-	135.0		15.85	–29.1/-	-	140.3	11.69	–31.5	104.3	13.19
PBAT/PLA_190	-	-	126.4	148.8	22.16	–29.3/-	-	141.2	12.23	–32.7	113.6	10.72
PBAT/PLA/3Gly_190	-	-		141.1	20.62	–29.9/-	-	139.0	12.04	–33.8	105.1	11.91
After 30 Days of Anaerobic Digestion												
PBAT/PLA_155	94.3	4.47	133.7	153.8	7.67	–31.6/60.8	123.1	152.7	13.38	–36.3/-	102.8	8.15
PBAT/PLA/3Gly_155	97.3	3.77	134.3	153.8	7.33	–28.5/56.5	127.6	152.5	9.29	–36.7/-	101.0	5.09
PBAT/PLA_190	97.8	3.89	134.4	155.1	6.60	–30.3/61.6	133.1	153.6	6.83	–37.8/-	101.5	6.13
PBAT/PLA/3Gly_190	96.0	2.61	133.4	153.1	8.91	–31.7/58.4	129.0	153.4	7.30	–37.3/-	102.4	5.57
After 58 Days of Anaerobic Digestion												
PBAT/PLA_155	97.1	3.55	131.7	152.0	9.8	–31.1/55.0	130.9	-	7.19	–36.9/-	104.6	7.22
PBAT/PLA/3Gly_155	89.7	1.45	130.7	-	7.5	–30.0/53.2	133.3	-	5.18	–36.6/-	104.5	7.24
PBAT/PLA_190	94.3	1.17	133.2	-	7.02	–31.5/46.4	133.0	-	6.03	–36.7/-	103.2	8.89
PBAT/PLA/3Gly_190	101.4	3.61	131.9	-	5.34	–32.5/53.8	131.8	-	4.76	–34.0/-	105.7	6.80
After 99 Days of Anaerobic Digestion												
PBAT/PLA_155	65.6/100.6	1.06/1.60	134.3	141.5/149.1	9.88	–32.1/53.3	130.7	-	8.11	–39.8/-	104.6	9.78
PBAT/PLA/3Gly_155	83.2	2.64	134.8	138.8	9.50	–30.4/43.6	131.1	-	6.77	–36.9/-	106.7	7.68
PBAT/PLA_190	104.6	3.77	135.4	-	8.29	–30.7/52/1	133.4	-	8.12	–36.1/-	104.3	8.08
PBAT/PLA/3Gly_190	67.2/102.8	5.75	133.1	147.5	8.34	–33.0/48.8	131.2	-	5.72	–37.0/-	105.3	8.02

a RC, rapid cooling; *T*_*g*1_ and *T*_*g*2_, glass transition temperature of PBAT and PLA, respectively; *T*_*cc*_, the maximum of the exothermic peak of the cold crystallization temperature; ΔΗ_*cc*_, cold crystallization enthalpy; *T*_*m*1_ and *T*_*m*2_, melting temperature of PBAT and PLA, respectively; Δ*H*_*m*_, melting enthalpy; *T*_*c*_, crystallization temperature; ΔΗ_*c*_, crystallization enthalpy; *, *T*_*m*_ PBAT and *T*_*cc*_ PLA regions may overlap.

In the DSC thermogram during the first heating run, showing the thermal history of the specimens, a broad endothermic melting effect was observed for all specimens before and after irradiation. However, the exothermic thermal effect of cold crystallization that occurred in the specimens before irradiation was not observed after irradiation. This indicates a greater order of the material after irradiation, which also indicates self-chain/cross-linking.

The endothermic effects for the PLA component of the PBAT/PLA blend for all specimens did not change significantly, while in the case of the PBAT component of both specimens printed at 155 °C, the temperature range is slightly higher than the melting temperature (*T*_*m*_) of the specimens before UV exposure as well as the enthalpy, which has doubled. In contrast, in the case of the PBAT component of both specimens printed at 190 °C, the temperature range is slightly lower, and the enthalpy of melting has slightly decreased. In the DSC curves of the PBAT/PLA/3Gly_155 specimen, in addition to the main melting effect, an additional endothermic effect was found over a wide temperature range below the *T*_*m*_ value of the specimen before UV exposure. The occurrence of an additional melting effect is associated with the melting of smaller crystallites with a poorly shaped structure. In these cases, there are broad endothermic melting effects of crystallites with a strongly disordered structure at a temperature range lower than the temperature corresponding to the melting effects of the specimen before UV exposure.^37^ This effect may indicate the degradation of the PLA component of the PBAT/PLA blend. This is also confirmed by the NMR analyses. For PBAT/PLA_155 and PBAT/PLA/3Gly_190, 1 mol·% of PLA loss was observed (see Table 4).

**Table 4:** Composition and Dyad Sequences Determined Using ^1^H NMR Based on ref 19 of PBAT/PLA-Based Specimens before and after UV Exposure, as well as after 77 and 97 Days of Aerobic Composting and after 30, 58, and 99 Days of Anaerobic Digestion^a^

specimen	PBAT/PLA_155 [mol·%]	PBAT/PLA/3Gly_155 [mol·%]	PBAT/PLA_190 [mol·%]	PBAT/PLA/3Gly_190 [mol·%]
Before Degradation				
PBAT/PLA	75/25	75/25	75/25	75/25
BT/BA	47/53	47/53	47/53	47/53
After UV Exposure				
PBAT/PLA	76/24	75/25	75/25	76/24
BT/BA	47/53	47/53	47/53	47/53
After 77 Days of Aerobic Composting				
PBAT/PLA	98/2	94/6	97/3	92/2
BT/BA	50/50	49/51	49/51	49/51
After 97 Days of Aerobic Composting				
PBAT/PLA	100/0	99/1	100/0	100/0
BT/BA	51/49	50/50	52/48	51/49
After 30 Days of Anaerobic Digestion				
PBAT/PLA	77/23	80/20	80/20	79/21
BT/BA	47/53	47/53	47/53	47/53
After 58 Days of Anaerobic Digestion				
PBAT/PLA	87/13	85/15	93/7	85/15
BT/BA	47/53	48/52	48/52	48/52
After 99 Days of Anaerobic Digestion				
PBAT/PLA	88/12	95/5	92/8	91/9
BT/BA	48/52	48/52	47/53	48/52

a BT, 1,4-butylene terephthalate units of PBAT; BA, 1,4-butylene adipate units of PBAT.

From the second heating run (see Table 3), it appears that irradiation has no pronounced influence on *T*_*g*_; however, a decrease in the *T*_*m*_ value during the second heating run may indicate self-chain/cross-linking.^36^ The specimens were recrystallized in the presence of self-chain/cross-links formed during UV exposure, which act as defect centers, limiting the mobility of the PBAT/PLA chains and causing a decrease in *T*_*m*_. Consequently, order decreases with increasing self-chain/cross-link density due to more limited mobility and conformational rearrangement of polymer chains to form crystals.

Radiation itself affects many polymer properties, i.e., it lowers *T*_*g*_, cold crystallization temperature (*T*_*cc*_), and *T*_*m*_. UV self-chain/cross-linking can, however, lead to an increase in these parameters, although it should be remembered that UV light has a limited depth of penetration.^38^ It was found that the UV exposure causes degradation of the PLA component (also lowering *T*_*m*_) and self-chain/cross-linking of the PBAT component of the blend, which was to be expected^14^ and which is reflected in the ambiguous DSC results (e.g., no *T*_*cc*_ but lower crystallization temperature (*T*_*c*_) after exposure for specimens obtained at a lower printing temperature). However, the thermal decomposition of PBAT/PLA-based specimens assessed by TGA (Table 5) indicates that, in addition to PLA degradation, its self-chaining/cross-linking also occurs.

**Table 5:** Thermogravimetric Parameters of PBAT/PLA-Based Specimens before and after UV Exposure, as well as after 77 and 97 Days of Aerobic Composting and after 30, 58, and 99 Days of Anaerobic Digestion^a^

	PLA	BT units	BA units
specimen	*T*_*max*_ [°C]	*T*_*max*_ [°C]	*T*_*max*_ [°C]
Before Degradation			
PBAT/PLA_155	321.6	401.6	413.4
PBAT/PLA/3Gly_155	321.7	402.4	414.3
PBAT/PLA_190	314.7	402.2	414.1
PBAT/PLA/3Gly_190	320.9	402.8	413.8
After UV Exposure			
PBAT/PLA_155	323.5	402.7	412.9
PBAT/PLA/3Gly_155	321.7	401.1	413.5
PBAT/PLA_190	325.4	401.5	414.3
PBAT/PLA/3Gly_190	321.5	402.2	414.5
After 77 Days of Aerobic Composting			
PBAT/PLA_155	-	402.4	ND
PBAT/PLA/3Gly_155	ND/ND	404.2	ND
PBAT/PLA_190	ND	401.5	ND
PBAT/PLA/3Gly_190	-	404.1	ND
After 97 Days of Aerobic Composting			
PBAT/PLA_155	-	401.2	ND
PBAT/PLA/3Gly_155	ND	395.4	-
PBAT/PLA_190	-	396.8	-
PBAT/PLA/3Gly_190	-	403.0	-
After 30 Days of Anaerobic Digestion			
PBAT/PLA_155	280.5	402.3	413.0
PBAT/PLA/3Gly_155	280.1	402.5	ND
PBAT/PLA_190	283.3/ND	399.6	412.1
PBAT/PLA/3Gly_190	288.5/ND	400.7	ND
After 58 Days of Anaerobic Digestion			
PBAT/PLA_155	281.3	401.3	ND
PBAT/PLA/3Gly_155	284.9/ND	400.1	412.5
PBAT/PLA_190		401.3	413.8
PBAT/PLA/3Gly_190	285.1/ND	400.5	ND
After 99 Days of Anaerobic Digestion			
PBAT/PLA_155	280.7	400.5	413.5
PBAT/PLA/3Gly_155	-	400.4	413.5
PBAT/PLA_190	ND	398.7	411.8
PBAT/PLA/3Gly_190	ND	400.3	413.7

a ND, No data/Not determined; *T*_max_, temperature at the maximum decomposition rate; BT, 1,4-butylene terephthalate units of PBAT; BA, 1,4-butylene adipate units of PBAT.

Thermal decomposition curves of PBAT/PLA-based specimens before and after UV exposure proceeded with multiple mass loss steps.^19^ Before degradation, the first step corresponds to the thermal decomposition of PLA with *T*_*max*_ ≈ 322 °C for specimens printed at 155 °C and for specimens printed at 190 °C, *T*_*max*_ ≈ 315 °C without oligopeptide and 321 °C with oligopeptide, while the decomposition of PBAT shows a higher thermal stability with two decomposition temperatures due to its aliphatic-aromatic structure with longer segments. For PBAT, one *T*_*max*_ is for 1,4-butylene terephthalate units^39^ with *T*_*max*_ ≈ 402 °C and *T*_*max*_ ≈ 403 °C for specimens printed in 190 °C with oligopeptide, the other *T*_*max*_ is for 1,4-butylene adipate units^40^ with *T*_*max*_ ≈ 413 °C for specimens printed in 155 °C and *T*_*max*_ ≈ 414 °C for the rest of the specimens (see Table 5). The subsequent decomposition steps correspond to the commercial additives introduced into the blend by the manufacturer.

The printing temperature of 190 °C slightly reduces the thermal stability of PLA, which is probably related to the slight molar mass loss when processed at such a high temperature.^41^ However, in the case of the oligopeptide-modified PBAT/PLA-based specimens printed at 190 °C, this decrease was significantly reduced. The oligopeptide in the polymer matrix acted as a plasticizer, like a lubricant, reducing frictional forces between the polymer chains, thereby minimizing thermal degradation by reducing interchain interactions and helping to maintain the structural integrity of the polymer.^42^

After UV exposure, the *T*_*max*_ value of PLA increased, and, for the PBAT/PLA_190 specimen with the lowest value before irradiation, it was *T*_*max*_ ≈ 325 °C. In contrast, the *T*_*max*_ of PBAT did not change. Self-chain/cross-linking promotes increased thermal stability of the polymer, making it more rigid and less prone to degradation. The oligopeptide in the polymer matrix acted as a plasticizer, making the material softer and more flexible by reducing its intermolecular forces. This opposing effect is due to the way self-chain/cross-linking and plasticization alter the molecular structure of the polymer, with self-chain/cross-linking promoting a more rigid and three-dimensional network and plasticization creating a more amorphous and flexible chain conformation.^43^ While PBAT, having higher thermal stability, is less susceptible to the effects of a small amount of oligopeptide plasticizer.

SEM examination of the surface of the PBAT/PLA-based specimens revealed greater roughness of the specimens after UV exposure in the form of a mosaic visible on the surface, indicating the formation of partially ordered areas.^44^ The surface of the PBAT/PLA-based specimens before UV exposure appears smoother, showing relatively uniform character and low surface crystallization (Figure 8).

**Figure 8:**
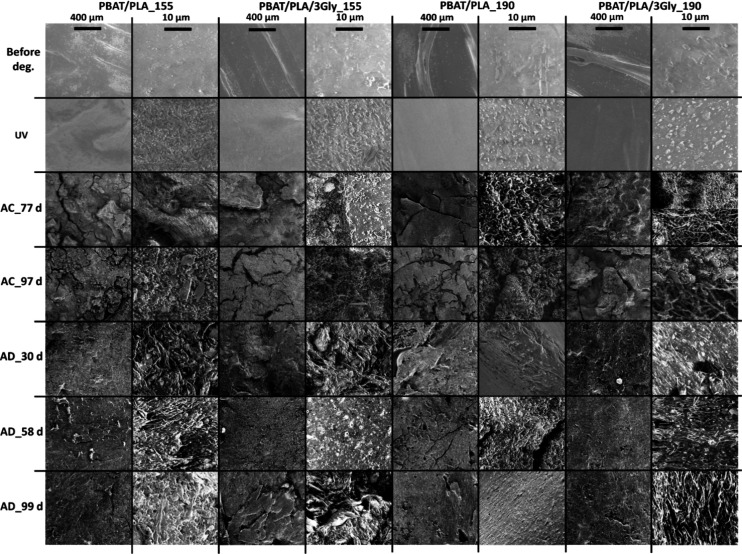
Representative SEM images of the surface of the PBAT/PLA-based specimens before degradation, after UV exposure as well as after 77 and 97 days of aerobic composting (AC) and after 30, 58, and 99 days of anaerobic digestion (AD) (magnification: 200 and 5000×).

On the one hand, exposure to UV radiation can lead to chain scission in the polymer amorphous domains.^45^ The resulting fragments can then rearrange and align, forming new crystalline structures. At the same time, the self-chaining and cross-linking that occur also lead to increased order.

### Aerobic Composting and Anaerobic Digestion

Aerobic composting of PBAT/PLA-based specimens was carried out under laboratory conditions for a period of 97 days, and anaerobic digestion for 99 days. The progress of specimens’ biodegradation was assessed on the basis of examination and failure analysis of the specimens, including both macroscopic and microscopic observations of their surfaces, and by monitoring changes in molecular structure and thermal properties throughout the experiments. PBAT/PLA-based specimens were removed from the rectors after 11 and 14 weeks of incubation in compost (77 and 97 days) and from anaerobic reactors after 1, 2, and 3 months (30, 58, and 99 days). The average temperature in the specimen reactors ranged from 55 ± 0.5 °C for PBAT/PLA/3Gly_155 specimens to 56 ± 0.5 °C for the other specimens, in the negative control reactors, 55 ± 0.4 °C, and in the positive control reactors (paper), 56 ± 0.4 °C. The average ambient temperature from mid-July to mid-October 2023 was 25 ± 2.3 °C. The average temperature in the anaerobic digestion incubator was 37 ± 1 °C.

Macroscopic visual assessment of the PBAT/PLA-based specimens during biodegradation revealed erosion in the form of cracking of all specimens during aerobic composting and flaking of the surface in the case of anaerobic digestion. After 99 days of anaerobic digestion, cracking was also observed, but only for the specimens with the oligopeptide (Figure 9). The presence of the oligopeptide in the form of a fine powder facilitated water access and specimen disintegration.^46^

**Figure 9:**
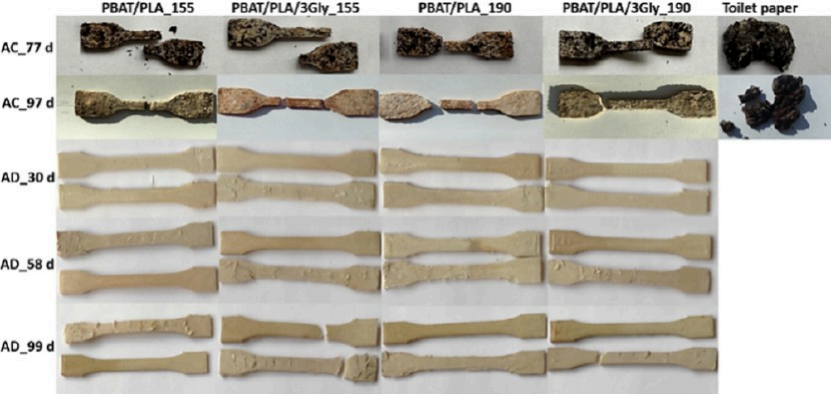
Representative images of PBAT/PLA-based specimens after 77 and 97 days of aerobic composting (AC) as well as after 30, 58, and 99 days of anaerobic digestion (AD).

In the specimens printed horizontally, the upper and lower layers had surfaces with distinct characteristics (Figure 10). This occurs because one layer is in direct contact with the 3D printer’s platform. However, after degradation and exposure to UV, these differences become blurred.

**Figure 10:**
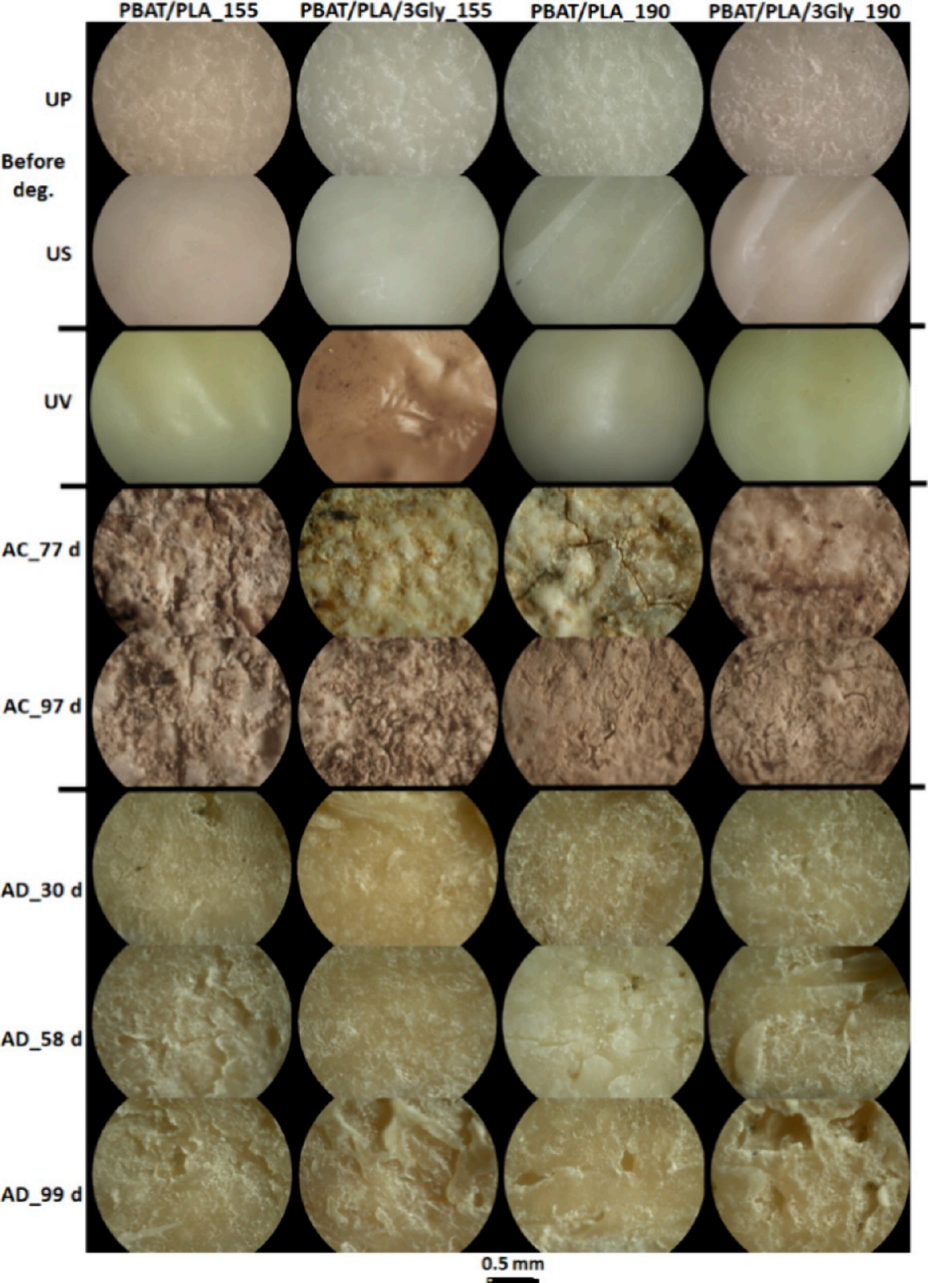
Representative optical microscope images of the surface of the PBAT/PLA-based specimens before degradation, after UV exposure, as well as after 77 and 97 days of aerobic composting (AC) and after 30, 58, and 99 days of anaerobic digestion (AD); UP, upper layer; US, underside layer (magnification: 100×).

Microscopic evaluation of the specimen surface during the aerobic composting process showed erosion resulting from water absorption and subsequent action of microorganisms, such as roughness and cracks of the surface of the PBAT/PLA-based specimens. After 77 days, deep cracks were visible on the surface. However, after 97 days, these cracks become shallower, which is due to the “licking” of the surface by microorganisms (Figure 10).

Anaerobic digestion of PBAT/PLA-based specimens resulted in holes and delamination/irregularities of the specimens’ surface visible after the 30 days as well as heterogeneous specimen surfaces and cracks visible after 58 days (Figure 10). After 58 days, inclusions were also visible, which could indicate water absorption and decomposition to lower molar mass degradation products (Figure 11).

**Figure 11:**
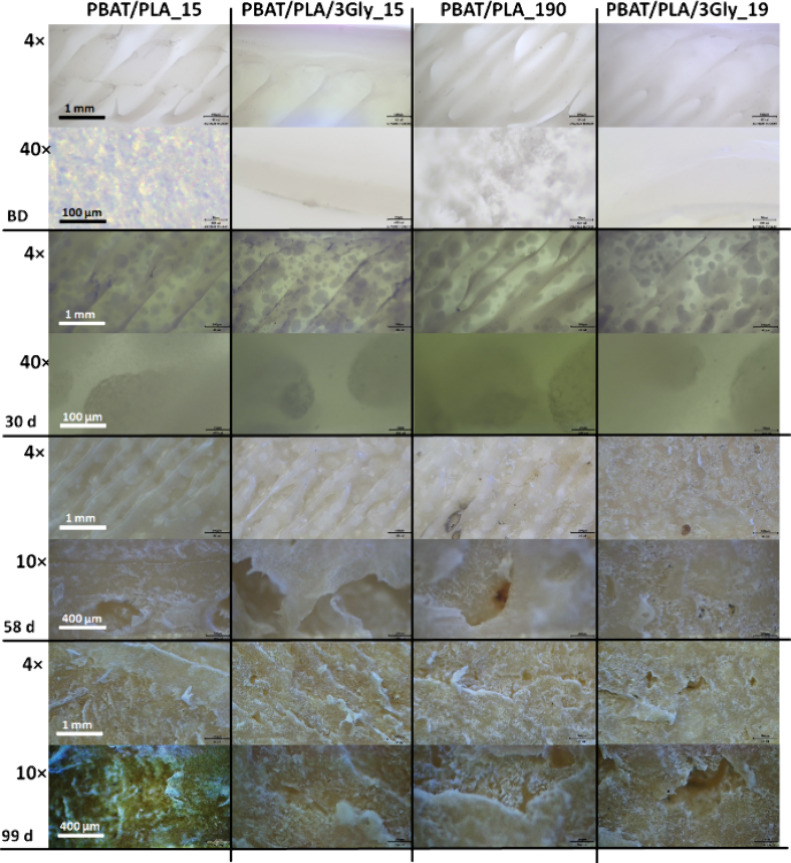
Representative optical microscope images of the surface of the PBAT/PLA-based specimens before (BD) and after 30, 58, and 99 days of anaerobic digestion (magnification: 4, 40, and 10×).

After 58 days of anaerobic digestion, depressions on the surface were visible at 10× magnification. Although the dynamics of biodegradation differed between aerobic and anaerobic conditions, the overall trend was the same.^47^

Microstructural surface analysis of the PBAT/PLA-based specimens by SEM (Figure 8) shows significant changes in the structure during and after both biodegradation experiments. After biodegradation, the biofilm formed by microorganisms on the surface is compact and covers most of the surface, which is visible at higher magnifications (5000× ; see Figure 8). The microbial biofilm visible on the surface of the PBAT/PLA-based specimens is shown in Figure 12.

**Figure 12:**
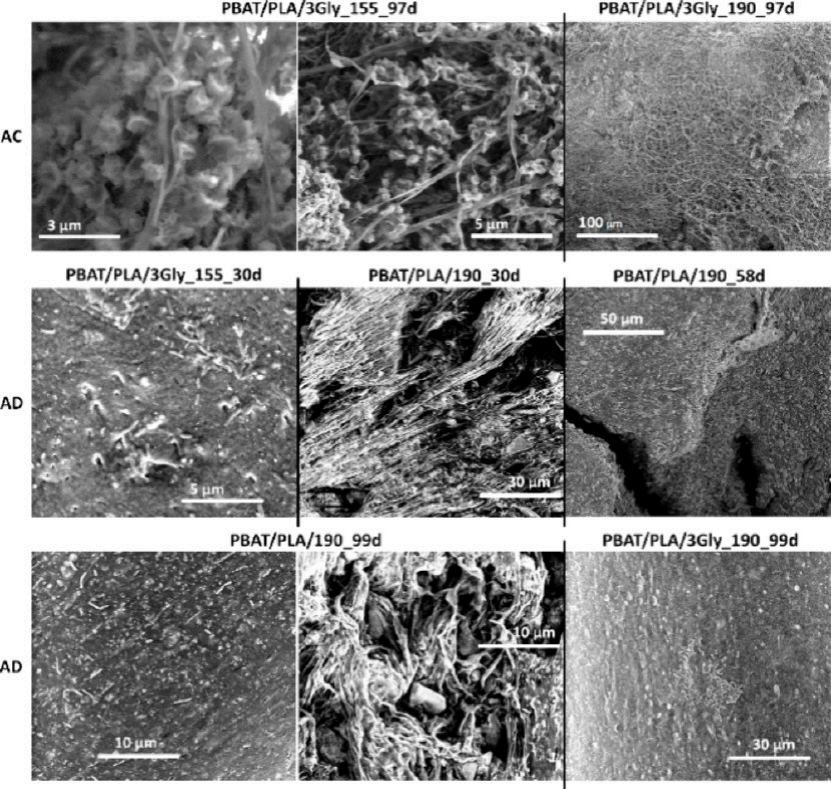
Representative SEM images of the surface of the PBAT/PLA/3Gly_155_97d and PBAT/PLA/3Gly_190_97d specimens after 97 days of aerobic composting (AC) as well as PBAT/PLA/3Gly_155_30d and PBAT/PLA_190_30d after 30 days, PBAT/PLA_190_58d after 58 days, and PBAT/PLA_190_99d and PBAT/PLA/3Gly_190_99d after 99 days of anaerobic digestion (AD) (magnification: 25 000, 10 000, 5000, 2500, 1000, and 500×).

From the beginning of the incubation period, both aerobic composting and anaerobic digestion resulted in a continuous decrease of the molar mass in all specimens, as illustrated in Figure 13.

**Figure 13:**
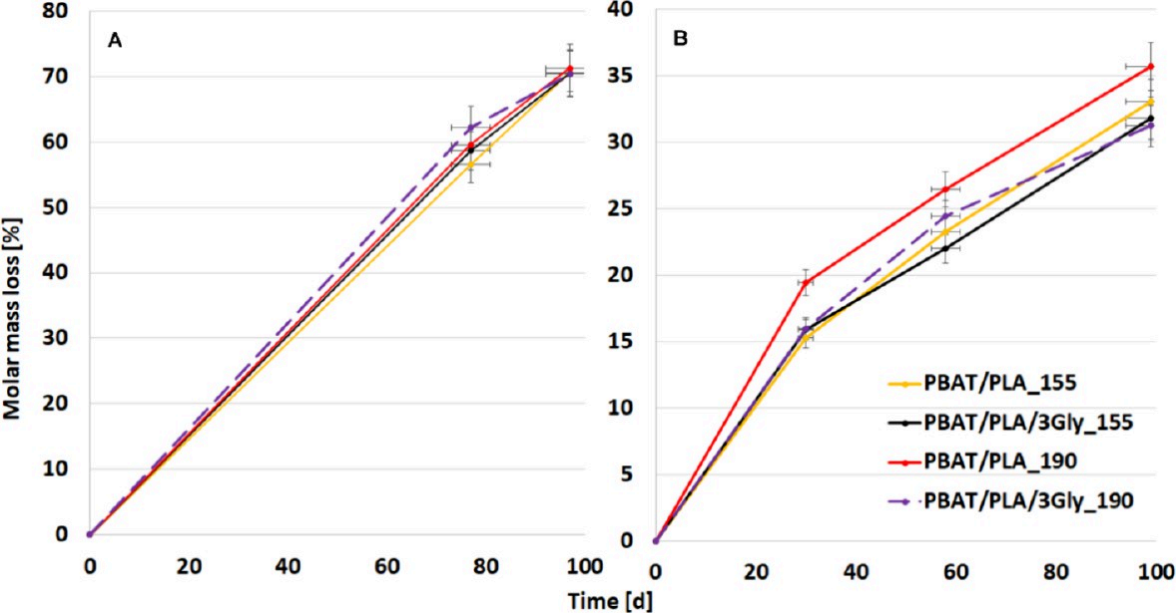
Molar mass loss from GPC of PBAT/PLA-based specimens as a function of incubation time (A) during aerobic composting and (B) anaerobic digestion.

In aerobic composting, there is also a significant loss of C (Figure 14).

**Figure 14:**
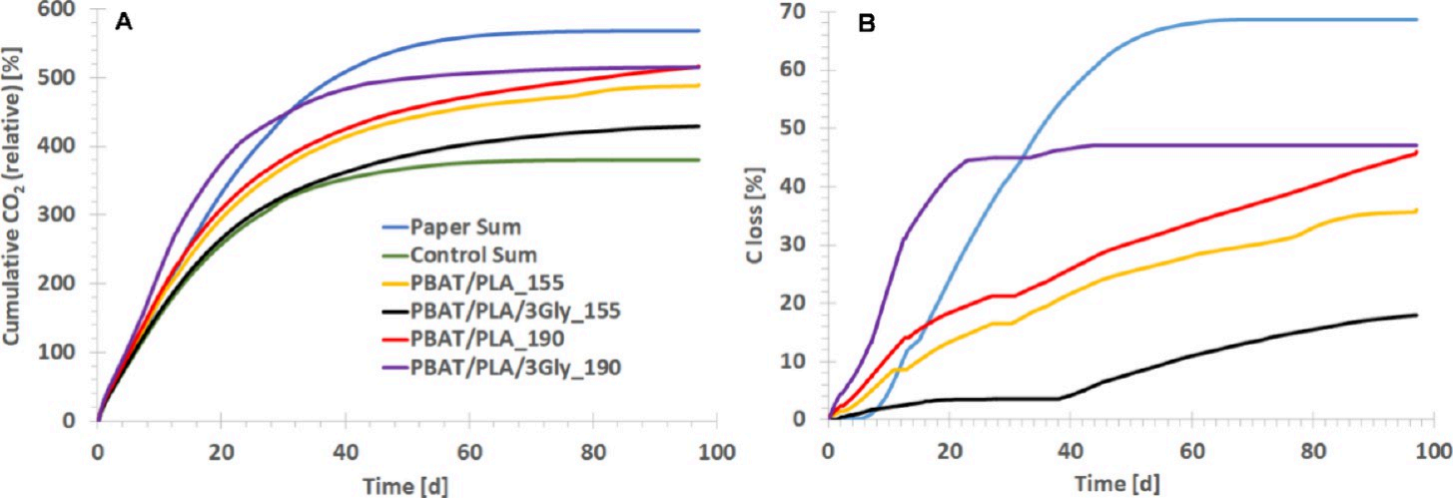
(A) Actual cumulative CO_2_ for PBAT/PLA-based specimens during 97 days of aerobic composting. (B) C loss for each PBAT/PLA-based dumbbell-shaped specimen during 97 days of aerobic composting. To determine this value, the CO_2_ from the negative control (compost without specimens) is subtracted from the CO_2_ from each treatment.

Figure 14A shows the plot representing the actual cumulative CO_2_ from each reactor and the average values for toilet paper (a cellulose-rich material) and the negative control (compost without specimens). For the positive controls from toilet paper, most of the release occurred during the first 60 days of incubation. As expected, the PBAT/PLA-based specimens had faster degradation than the negative control and reached the actual cumulative CO_2_ from 330 to 370% in the first 20 days of the experiment. Taking into account C loss during aerobic composting, the PBAT/PLA/3Gly_190 specimen showed the highest C loss of 47% in 97 days of incubation, while the lowest degradation was shown in the PBAT/PLA/3Gly_155 specimen (Figures 13 and 14). This was confirmed by ^1^H NMR analysis, which showed the presence of the PLA component both after 77 (6 mol·%) and 97 days (1 mol·%) of degradation of the PBAT/PLA/3Gly_155 specimen, whereas in the other specimens the PLA was 97–98% degraded after 77 days and 100% after 97 days. Interestingly, after 25 days of incubation, the C loss level reached 45%, which may indicate a high rate of degradation of the PLA component of the PBAT/PLA blend after 25 days of aerobic composting. The remaining PBAT degrades more slowly due to longer segments with aromatic units.^19^ The ^1^H NMR analysis showed the absence of the methine group signal of the PLA component of the blend after 97 days of aerobic composting, indicating its complete degradation except for the PBAT/PLA/3Gly_155 specimen, where 1 mol·% PLA remained (see Table 4). PBAT/PLA_190 and PBAT/PLA_155 specimens had slightly slower C loss of 46 and 36%, respectively, over 97 days of incubation, and with a constant rate of change of C loss. After 77 days of incubation in compost, C loss was 39 and 32%. The PBAT/PLA/3Gly_155 specimen had the slowest C loss of 18% after 97 days. The smallest C loss of PBAT/PLA/3Gly_155 (see Figure 14) may be since the average temperature in the specimen reactor was lower by 1 °C than in the other reactors. All four specimens have a similar molar mass loss from 57% for PBAT/PLA_155 to 62% for PBAT/PLA/3Gly_190 after 77 days of incubation, and almost the same 70 (with triglycine) and 71% (without triglycine) after 97 days of incubation. Comparable discrepancies in the correlation of polymer mass loss and CO_2_ release during aerobic composting conducted by other authors led to the conclusion based on a linear regression model that there was no straightforward correlation between CO_2_ generation and dry mass loss, from which it can be concluded that a similar trend also applies to molar mass.^48^ Additionally, during composting and, especially, during anaerobic digestion, various degradation mechanisms can occur. In fact, in addition to biodegradation, especially in the case of PLA, which mainly undergoes hydrolytic degradation in the environment, hydrolysis can simply occur.^49^ This changed the pattern of C loss with the overall degradation rate. Toilet paper used as a positive control shows the highest degradation rate as it mineralizes to 69% within 97 days of aerobic composting. The extent of biodegradation of the positive controls in the aerobic composting and anaerobic digestion experiments shows that conditions were conducive to aerobic and anaerobic degradation and confirms that the methods used worked as intended. However, although 86% of the cellulose (positive control) was mineralized within 99 days of anaerobic digestion (data not shown), the other PBAT/PLA-based specimens tested showed no significant loss of C, indicating degradation, although the loss of molar mass was significant. Therefore, C loss results for these specimens are not presented.

Taking into account molar mass loss during aerobic composting, for specimens 3D printed at 190 °C, this with the triglycine degrades the fastest, followed by the one without triglycine. The same effect is observed for the specimens 3D printed at a lower temperature of 155 °C, where PBAT/PLA_155 has the lowest degradation rate. The degradation rate trend of aerobic composting is as follows: PBAT/PLA/3Gly_190 > PBAT/PLA_190 > PBAT/PLA/3Gly_155 > PBAT/PLA_155. On the other hand, during anaerobic digestion, the fastest degradation was for the PBAT/PLA_190 specimen with 36% of molar mass loss in 99 days of incubation, which is 50% lower compared to the composting process. PBAT/PLA-based specimens without the oligopeptide degraded faster, with 36 and 33% molar mass loss and over 99 days of incubation for 190 and 155 °C printing temperatures, respectively, followed by specimens with the oligopeptide at 32 and 31% for 155 and 190 °C printing temperatures. This may be since glycine is a hydrophobic amino acid and may retard water access to the polymer matrix. The degradation rate trend of anaerobic digestion is as follows: PBAT/PLA_190 > PBAT/PLA_155 > PBAT/PLA/3Gly_190 > PBAT/PLA/3Gly_155. Interestingly, for both degradation processes, the molar mass loss in the first stage (up to 77 and 58 days of degradation in compost and anaerobic conditions, respectively) is greater for specimens printed at 190 °C (more orderly). Processing of PBAT/PLA-based specimens at 190 °C leads to a greater ordering of the material (see ΔΗ_*cc*_ and ΔΗ_*m*_, Table 3), which is also slightly increased by the addition of an oligopeptide as a crystallization nucleus. It is known that the addition of a fine powder can cause a nucleation effect. For the reason that the PBAT/PLA blend is immiscible, and as the order increases, the immiscibility also increases, leading to greater porosity and making it easier for water to penetrate deeper into the amorphous areas in the matrix, accelerating degradation at the initial stage.^50^ Previous study^13^ confirmed that in the case of the PBAT/PLA/3Gly_190 specimen, the SEM fracture morphology reflects slightly higher roughness of the fracture surface of the PBAT/PLA/3Gly printed at a higher temperature.

The loss of molar mass is consistent with the observed mass loss of the specimens, while the highest water absorption, conversely, is the highest in the case of specimens with oligopeptide (Figure 15). This higher absorption may also be due to the higher order/immiscibility in the presence of the oligopeptide, which facilitates the access of water.^50^

**Figure 15:**
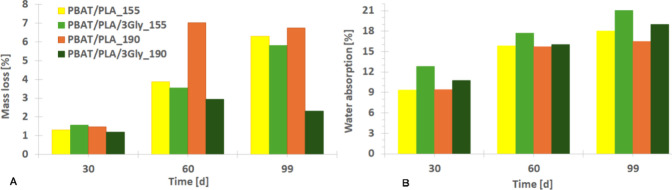
(A) Mass loss of PBAT/PLA-based specimens and (B) water absorption from sludge as a function of the incubation time during anaerobic digestion.

Thermal decomposition curves after aerobic composting proceeded with multiple mass loss steps; however, these stages were fewer than in the specimens before degradation due to the degradation first of the PLA blend component and then the 1,4-butylene adipate units of the PBAT copolymer (see Table 5). The first step of mass loss refers to the thermal degradation of the PLA residue, where its *T*_*max*_ could no longer be determined, and the next step refers to the thermal degradation of the components of the PBAT copolymer. The stability of 1,4-butylene terephthalate units of the PBAT copolymer decreases only with a significant molar mass loss after 97 days of aerobic composting. The situation is slightly different in the case of anaerobic digestion. PLA degrades more slowly, and a clear decrease in its *T*_*max*_ = 280–289 °C is seen, indicating a decrease in the molar mass of the degraded PLA. However, together with the 1,4-butylene adipate units, the 1,4-butylene terephthalate units were also degraded from the beginning. Thus, despite the generally slower anaerobic degradation, the degradation of the 1,4-butylene terephthalate units is inherently more intense at the beginning of the process than in aerobic composting. The TGA results (Table 5) were consistent with the analysis of the ^1^H NMR spectra (Table 4). After biodegradation, in the case of aerobic composting of PLA, a component of the blend, it was practically a complete absence of signals from PLA. Only in the case of PBAT/PLA/3Gly_155, 1 mol·% remained and *T*_*max*_ was not determined for PLA after 97 days, while in the case of anaerobic digestion, it was from 5 mol·% (PBAT/PLA/3Gly_155, no *T*_*max*_ for PLA) through 8 and 9 mol·% (for specimens printed in 190 °C, *T*_*max*_ not determined for PLA) and 12 mol·% (PBAT/PLA_155, *T*_*max*_ = 281 °C for PLA).

### Cytotoxicity Assessment

Advanced (bio)­degradable materials are considered a suitable option to minimize the environmental impact of polymers. The important requirement for a (bio)­degradable polymer to be used in medical applications is its biocompatibility, supporting the attachment and proliferation of cells. Therefore, the cytocompatibility of PBAT/PLA-based specimens and, for comparison, PBAT/PLA filaments without (PBAT/PLA) and with oligopeptide (PBAT/PLA/3Gly) were assessed using the MTT cytotoxicity assay. MTT assay is a gold standard method for assessing cell viability and proliferation, based on the ability of live functioning mitochondria to oxidize MTT reagent, which produces a typical blue-violet crystal product. The absorbance values obtained at 570 nm by solubilizing the crystals with DMSO can be directly correlated to the number of viable cells. As mentioned in the Materials and Methods section, four different oncogenic human cell lines from different tissue sources with different proliferation characteristics were used as models for this assay, as they have varying rates of proliferation and sensitivity to *in vitro* treatments. The cancer cell lines tend to grow faster than normal human fibroblast cell lines and respond quickly to any potential cytotoxicity induced by the polymers. Here it was used a direct method where the samples were semi-immersed, not touching the bottom of the plate or interfering with the monolayer of cells. Therefore, considering the morphology of the dead cells (Figure 16), any cytotoxicity observed must be related to the leaching of cytotoxic constituents from the test samples. Considering the cytotoxicity observed in this direct method, it can be concluded that the results will be similar for the indirect method. Figure 16 shows the microscopic images of all four cell lines grown with or without the PBAT/PLA-based specimens and PBAT/PLA filament strips.

**Figure 16:**
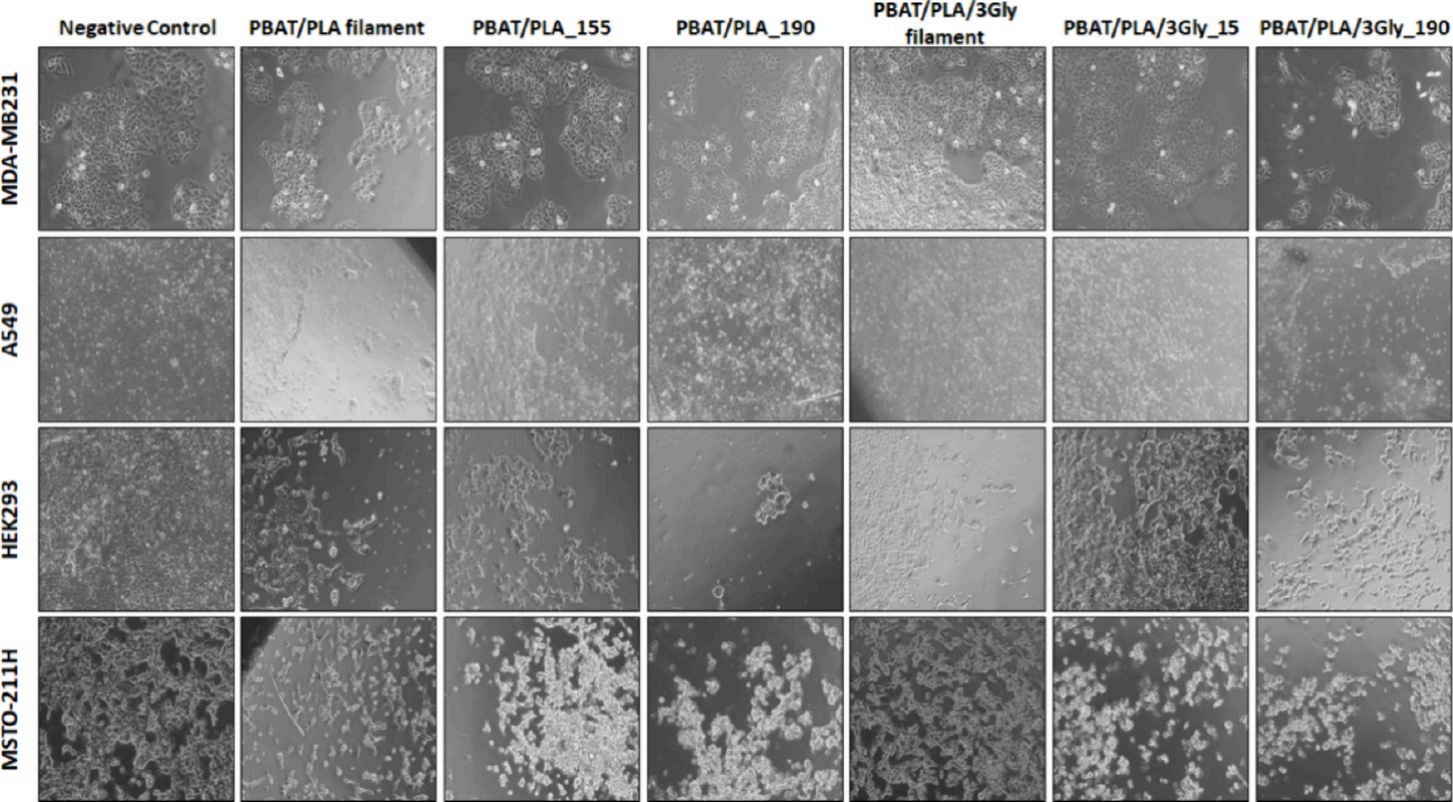
Representative phase contrast photomicrographs of cells after 72 h of culture with the PBAT/PLA-based specimens and PBAT/PLA filament without (PBAT/PLA) and with oligopeptide (PBAT/PLA/3Gly) (magnification: 10×).

The percentage of cell viability obtained from the MTT assay is shown in the bar chart in Figure 17.

**Figure 17:**
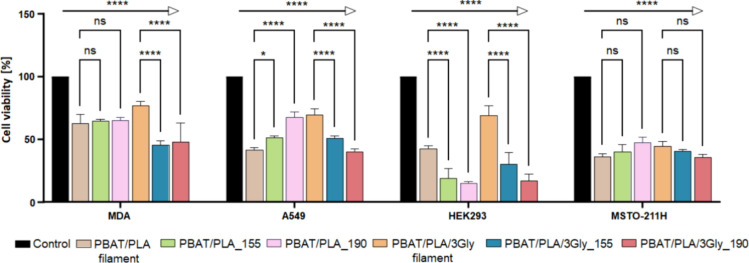
Cytocompatibility of the PBAT/PLA-based specimens and PBAT/PLA filament without (PBAT/PLA) and with oligopeptide (PBAT/PLA/3Gly). Bar chart indicates the percentage cell viability obtained by MTT assay after 72 h of culture in comparison to control cells grown without specimen strips; * *p* < 0.05, ****p < 0.0001; ns, no significance.

All PBAT/PLA-based specimens and both filaments clearly induced some form of statistically significant (*p* < 0.001) cytotoxicity to all cell lines tested. The cytotoxicity was dependent on the cell line, where HEK293 and MSTO were found to be the most sensitive cells, reaching cell death of up to 85%, and MDA had better viability, with about 50% of cells viable after 3 days. Although all specimens had exhibited significant cytotoxicity toward all cell lines, further statistical analysis between the sample groups suggests that the PBAT/PLA filament with oligopeptide had better viability in all four cell lines tested when compared to the PBAT/PLA filament without oligopeptide (Figure 18).

**Figure 18:**
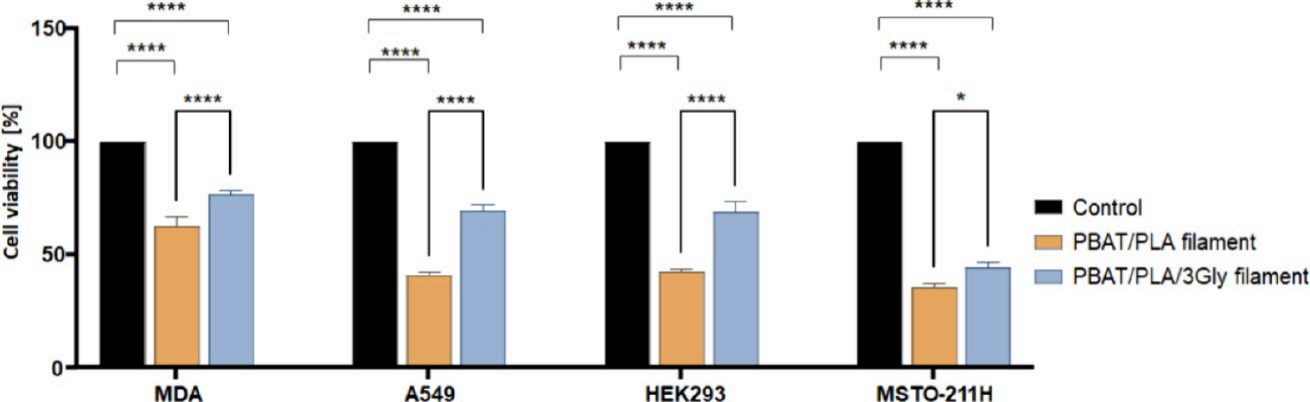
Comparison of the cytocompatibility of PBAT/PLA and PBAT/PLA/3Gly filaments. Bars indicate the percentage of cell viability obtained from MTT assay after 72 h of culture compared to cells grown without specimen strips; * *p* < 0.05, ****p < 0.0001.

Further comparisons of the phase contrast microscopic images of the specimens indicated that both processing temperatures showed signs of cytotoxicity in comparison to filaments. This was more obvious in HEK293 and MSTO cell lines than in the MDA and A549 cells. Similarly, the morphological images of the PBAT/PLA/3Gly filament indicated the same trend, where the PBAT/PLA-based specimens with oligopeptide printed at 155 and 190 °C had induced significant cytotoxicity in all four cells, and where the 190 °C specimens had much higher cytotoxicity. This is further confirmed by the MTT data, where PBAT/PLA-based specimens with oligopeptide printed at 155 and 190 °C showed a statistically significant reduction in cell viability in line with the temperature increase. The PBAT/PLA/3Gly_190 specimens had the lowest cell viability compared to PBAT/PLA/3Gly_155 and PBAT/PLA/3Gly filament (*p* < 0.001). On the other hand, it was observed that the PBAT/PLA-based specimens with oligopeptide printed at 155 and 190 °C, in comparison to the PBAT/PLA filament, did not show any statistically significant reduction in cell viability in three out of four cell lines tested (except HEK293). This is different from the observations of microscopic images, indicating that although morphological changes were seen, the cells were still viable under the MTT assay.

Filament with an oligopeptide has the highest cell viability in most cases (except for the MSTO cell line). Interestingly, PBAT/PLA-based specimens with an oligopeptide tend to have the lowest cell viability. Processing the PBAT/PLA filament with oligopeptide in the extruder at a temperature of around 120 °C did not result in as much change in material properties as subsequent reprocessing during printing at 155 and 190 °C.

Overall, the results showed that printing of PBAT/PLA specimens without triglycine at higher temperatures did not affect the cytocompatibility of these specimens. However, when the PBAT/PLA/3Gly specimens were printed at 155 and 190 °C, the cytocompatibility was significantly compromised. Therefore, it can be assumed that higher temperatures affected the triglycine marker in these specimens and potentially leached triglycine into the medium, leading to cell toxicity. Hence, it was tested whether the free triglycine has any cytotoxicity to the cells. Figure 19 shows that even very high concentrations (1 mg/mL) of triglycine did not have any significant effect on the cell viability of MDA and MSTO cell lines. Therefore, further studies are required to determine the effect of the temperature on the cytocompatibility of PBAT/PLA and PBAT/PLA/3Gly matrices.

**Figure 19:**
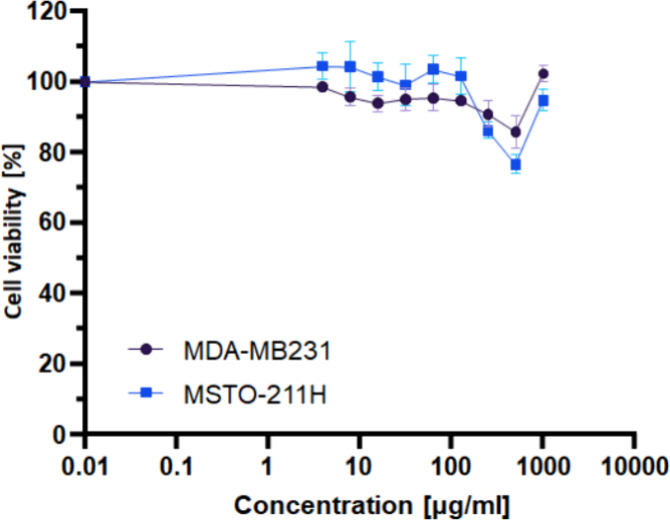
Cytotoxicity of triglycine in the MDA and MSTO cell lines. Cell viability curves show the dose-related response of two cell lines in percentage cell viability obtained from the MTT assay after 72 h of exposure to varying concentrations of triglycine.

Glycine is generally resistant to high temperatures and oxygen conditions and was not affected by heat treatment within the temperature range used during processing. Thermal analysis of glycine showed that its decomposition occurs not until 250 °C. Glycine is also an exogenous inhibitor and can inhibit the formation of some harmful substances.^51–53^ Previous studies have shown that PBAT/PLA films had higher cytocompatibility compared to poly­(l-lactide) (PLLA).^5^ The neat PLLA film after biodegradation showed even lower cell viability because degradation of PLLA leads to acidification of the environment. In other studies, the incorporation of nanohydroxyapatite into PLLA resulted in neutralization of the acidic environment and reduced its negative impact.^54^ All of this may lead to the conclusion that thermal degradation products of the PBAT/PLA matrix could cause a cytotoxic effect.

## Conclusions

Green polymer/oligopeptide systems of PBAT/PLA with triglycine dispersed in the polymer matrix were prepared and tested under UV irradiation, composting, and anaerobic digestion environments. Triglycine was used as a molecular label for the PBAT/PLA polymer in the context of data storage. The encoded information, as an exemplary number, 7, was decoded using a mass spectrometry analysis of the supernatant after extraction of the polymer films. The study highlights the high sensitivity of information encoded in the molecular structure of oligopeptides, which can be effectively analyzed using mass spectrometry techniques like ESI/TIMS-Q-TOF. However, this technique has limitations compared to the MALDI-TOF/TOF presented in a previous publication.^5^ The inability to label the amino acid sequence based on the fragment mass spectrum for short amino acid sequences like the GGG oligopeptide was analyzed in this work. Nevertheless, the ion mobility dimension of ESI/TIMS-Q-TOF improves the identification accuracy, sensitivity, and reproducibility compared to methods measuring *m*/*z* values only. UV exposure altered the *T* *_cc_* and *T*_*m*_ and caused self-chaining/cross-linking of PBAT/PLA-based specimens. DSC analysis showed that UV exposure causes degradation of the PLA component and self-chain/cross-linking of the PBAT component of the blend, which was to be expected, but the thermal decomposition of the PBAT/PLA-based specimens assessed by TGA indicates that self-chain/cross-linking of PLA occurs in addition to its degradation. After UV exposure, the *T*_*max*_ value of PLA increased, while that of PBAT remained unchanged, suggesting that self-chain/cross-linking improved the thermal stability of PLA. On the other hand, the triglycine also acted as a plasticizer. Due to its higher thermal stability, PBAT was less affected by small additions of oligopeptide. The SEM analysis reveals major surface changes during biodegradation experiments, with the formation of a dense biofilm indicating microbial activity. The findings suggest that specimens printed at 190 °C have a higher degradation rate than those printed at lower temperatures, likely due to increased immiscibility, resulting in higher porosity, which facilitated easier water penetration. In aerobic composting, in both NMR and TGA, the highest loss of PLA and 1,4-butylene adipate units of PBAT from the blend was observed for specimens printed at 190 °C without oligopeptide (PBAT/PLA_190), and the lowest loss of PLA was observed for specimens printed at 155 °C with oligopeptide (PBAT/PLA/3Gly_155). Conversely, the degradation rate and molar mass loss were the highest (62% after 77 days) for specimens printed at 190 °C with an oligopeptide (PBAT/PLA/3Gly_190) and the lowest (57% after 77 days) for specimens printed at 155 °C without an oligopeptide (PBAT/PLA_155). However, the loss of molar mass at 97 days was already comparable for all specimens—70% with oligopeptide and 71% without oligopeptide (from GPC). In less humid environments, the hydrophobic oligopeptide may also partially act as a moisture repellent,^55^ especially when the degraded polymer matrix contains more oligopeptide, which may degrade more slowly. During anaerobic digestion, the loss of PLA and 1,4-butylene adipate units of PBAT from the blend, degradation rate, and loss of molar mass after 99 days were the highest for PBAT/PLA_190 (NMR and GPC). Oligopeptide-containing samples disintegrated more as they also absorbed more water (an effect of immiscibility due to higher ordering and the presence of oligopeptide as a fine powder, which facilitates water penetration into the polymer matrix and increases the extent of degradation in a more humid environment). GPC analysis indicated that anaerobic degradation proceeds at approximately 50% of the rate of composting. The measurements of released gases and C losses for each PBAT/PLA-based specimen were not at a sufficiently reliable level to interpret these results. Cytotoxicity tests showed that processing of the PBAT/PLA matrix with an oligopeptide at above 120 °C reduced cell viability. PBAT/PLA-based specimens were found to be cytotoxic and therefore not biocompatible (statistics showed significance between groups, indicating cytotoxicity), potentially limiting their application.
